# Ethylene Promotes Hypocotyl Growth and HY5 Degradation by Enhancing the Movement of COP1 to the Nucleus in the Light

**DOI:** 10.1371/journal.pgen.1004025

**Published:** 2013-12-12

**Authors:** Yanwen Yu, Juan Wang, Zhijin Zhang, Ruidang Quan, Haiwen Zhang, Xing Wang Deng, Ligeng Ma, Rongfeng Huang

**Affiliations:** 1 Biotechnology Research Institute, Chinese Academy of Agricultural Sciences, Beijing, China; 2 National Key Facility of Crop Gene Resources and Genetic Improvement, Beijing, China; 3 Department of Molecular, Cellular, and Developmental Biology, Yale University, New Haven, Connecticut, United States of America; 4 College of Life Science, Capital Normal University, Beijing, China; University of North Carolina at Chapel Hill, United States of America

## Abstract

In the dark, etiolated seedlings display a long hypocotyl, the growth of which is rapidly inhibited when the seedlings are exposed to light. In contrast, the phytohormone ethylene prevents hypocotyl elongation in the dark but enhances its growth in the light. However, the mechanism by which light and ethylene signalling oppositely affect this process at the protein level is unclear. Here, we report that ethylene enhances the movement of CONSTITUTIVE PHOTOMORPHOGENESIS 1 (COP1) to the nucleus where it mediates the degradation of LONG HYPOCOTYL 5 (HY5), contributing to hypocotyl growth in the light. Our results indicate that HY5 is required for ethylene-promoted hypocotyl growth in the light, but not in the dark. Using genetic and biochemical analyses, we found that HY5 functions downstream of ETHYLENE INSENSITIVE 3 (EIN3) for ethylene-promoted hypocotyl growth. Furthermore, the upstream regulation of HY5 stability by ethylene is COP1-dependent, and COP1 is genetically located downstream of EIN3, indicating that the COP1-HY5 complex integrates light and ethylene signalling downstream of EIN3. Importantly, the ethylene precursor 1-aminocyclopropane-1-carboxylate (ACC) enriched the nuclear localisation of COP1; however, this effect was dependent on EIN3 only in the presence of light, strongly suggesting that ethylene promotes the effects of light on the movement of COP1 from the cytoplasm to the nucleus. Thus, our investigation demonstrates that the COP1-HY5 complex is a novel integrator that plays an essential role in ethylene-promoted hypocotyl growth in the light.

## Author Summary

It is well known that light suppresses hypocotyl growth in seedlings, while the phytohormone ethylene and its precursor 1-aminocyclopropane-1-carboxylate (ACC) enhance hypocotyl growth in the light. However, the mechanism by which light and ethylene oppositely affect this process at the protein level is unclear. Here, we demonstrate that ethylene enhances the movement of CONSTITUTIVE PHOTOMORPHOGENESIS 1 (COP1) to the nucleus where it promotes the degradation of LONG HYPOCOTYL 5 (HY5) in the light, contributing to hypocotyl growth. Our data indicate that HY5 is required for ethylene-promoted hypocotyl growth in the light, but not in the dark. Using genetic and biochemical analyses, we found that HY5 functions downstream of ETHYLENE INSENSITIVE 3 (EIN3) during ethylene-promoted hypocotyl growth. Further, the regulation of HY5 stability by ethylene is COP1-dependent, and COP1 is genetically located downstream of EIN3, indicating that the COP1-HY5 complex integrates light and ethylene signalling downstream of EIN3. Importantly, ACC enriched the nuclear localisation of COP1 in an EIN3-dependent manner in the presence of light, suggesting that ethylene rescued the effects of light on the movement of COP1 from the cytoplasm to the nucleus. Thus, our investigation shows that the COP1-HY5 complex is a novel integrator that plays an essential role in ethylene-promoted hypocotyl growth in the light.

## Introduction

The phytohormone ethylene plays significant roles in many developmental processes and stress responses in plants. Molecular and genetic analyses have revealed a linear signalling pathway, which is initiated by ethylene perception at the endoplasmic reticulum membrane, resulting in transcriptional regulation in the nucleus [1]–[3]. The ethylene receptors ETHYLENE RESPONSE 1 (ETR1), ETHYLENE RESPONSE SENSOR 1 (ERS1), ETR2, ERS2, and ETHYLENE INSENSITIVE 4 (EIN4) are members of a family of two-component His protein kinase receptors that negatively affect ethylene signaling [4], [5]. In the absence of ethylene, these receptors directly suppress the ethylene response by interacting with a Raf-like mitogen-activated protein kinase kinase kinase family protein, CONSTITUTIVE TRIPLE RESPONSE 1 (CTR1) [6]–[8]. This negative regulator interacts with and directly phosphorylates the cytosolic C-terminal domain of EIN2 in *Arabidopsis* [9]. EIN2 is a central positive regulator of ethylene signalling that is localised to the endoplasmic reticulum membrane through its N-terminal domain [10]. The phosphorylation of EIN2 by CTR1 prevents EIN2 from signalling in the absence of ethylene, whereas the inhibition of CTR1 upon ethylene perception is a signal for cleavage and translocation of EIN2 from the cytoplasm to the nucleus [9], [11]–[13]. The transcription factors EIN3 and EIN3-LIKE 1 (EIL1) act downstream of EIN2, which is required for the ethylene-induced stabilisation of EIN3/EIL1 [14], [15]. EIN3 further activates the expression of ethylene-responsive genes in different physiological processes [15]–[21].

When emerging from the soil, newly etiolated *Arabidopsis thaliana* seedlings display long hypocotyls, apical hooks, and closed cotyledons. Exposure to light inhibits hypocotyl growth and promotes the greening and expansion of the cotyledons and leaves [22]. During this processes, light modulates multiple hormonal pathways, including those involving gibberellins, abscisic acid, auxin, brassinosteroids, cytokinins, and ethylene, to regulate these developmental changes [23]–[32]. Increasing evidence suggests that gibberellins and cytokinins regulate the accumulation of the light signalling component LONG HYPOCOTYL 5 (HY5), a basic leucine zipper (bZIP) transcription factor that acts downstream of the light signal and positively regulates the transcription of light-induced genes [33], [34]. Light promotes the accumulation of HY5 protein by inhibiting the accumulation of CONSTITUTIVELY PHOTOMORPHOGENIC 1 (COP1) in the nucleus [35], [36]. Furthermore, HY5 reportedly promotes photomorphogenesis, in part by modulating auxin, cytokinin, and gibberellins signalling [32], , revealing the integration of light and phytohormone signalling. In addition, a recent study reported that EIN3/EIL1 cooperate with phytochrome interacting factor 1 (PIF1) and COP1 to optimise the de-etiolation of *Arabidopsis* seedlings and demonstrated that ethylene plays a key role in the establishment of green seedlings upon exposure to light [19]. Importantly, ethylene also inhibits *Arabidopsis* hypocotyl elongation in the dark, whereas ethylene and its precursor 1-aminocyclopropane-1-carboxylate (ACC) increase hypocotyl elongation in light-grown *Arabidopsis* seedlings [24], [39], [40], and EIN3 transcriptionally activates two contrasting pathways: the PIF3-dependent growth-promoting pathway and an ethylene response factor 1 (ERF1)-mediated growth inhibiting pathway, to fine-tune ethylene-promoted hypocotyl growth in the light [39]. Moreover, ethylene regulates the biosynthesis, transport, and distribution of IAA during light-mediated hypocotyl growth, dependent on the effect of COP1 on gene transcription downstream of EIN3 [29], [40]. These findings indicate the regulation of seedling growth by ethylene-light interactions; however, it remains unclear how the ethylene and light signalling pathways are integrated at the protein level to achieve the conserved regulation of plant hypocotyl growth.

In this study, we found that ethylene enhances the movement of COP1 to the nucleus to degrade HY5 in the light, revealing that the COP1-HY5 complex is a novel integrator of light-ethylene interactions during hypocotyl growth. Our data show that ethylene regulates hypocotyl growth by reducing HY5 protein levels. More importantly, ethylene-promoted hypocotyl elongation was inhibited in the absence of COP1 function, and COP1 was found to be required for HY5 stability. Further, ACC affected the EIN3-dependent shuttling of COP1 between the nucleus and cytoplasm. These results reveal that the light-regulated COP1-HY5 cascade is involved in ethylene-promoted hypocotyl growth.

## Results

### HY5 mediates ethylene-promoted hypocotyl elongation in the light

**Figure 1:**
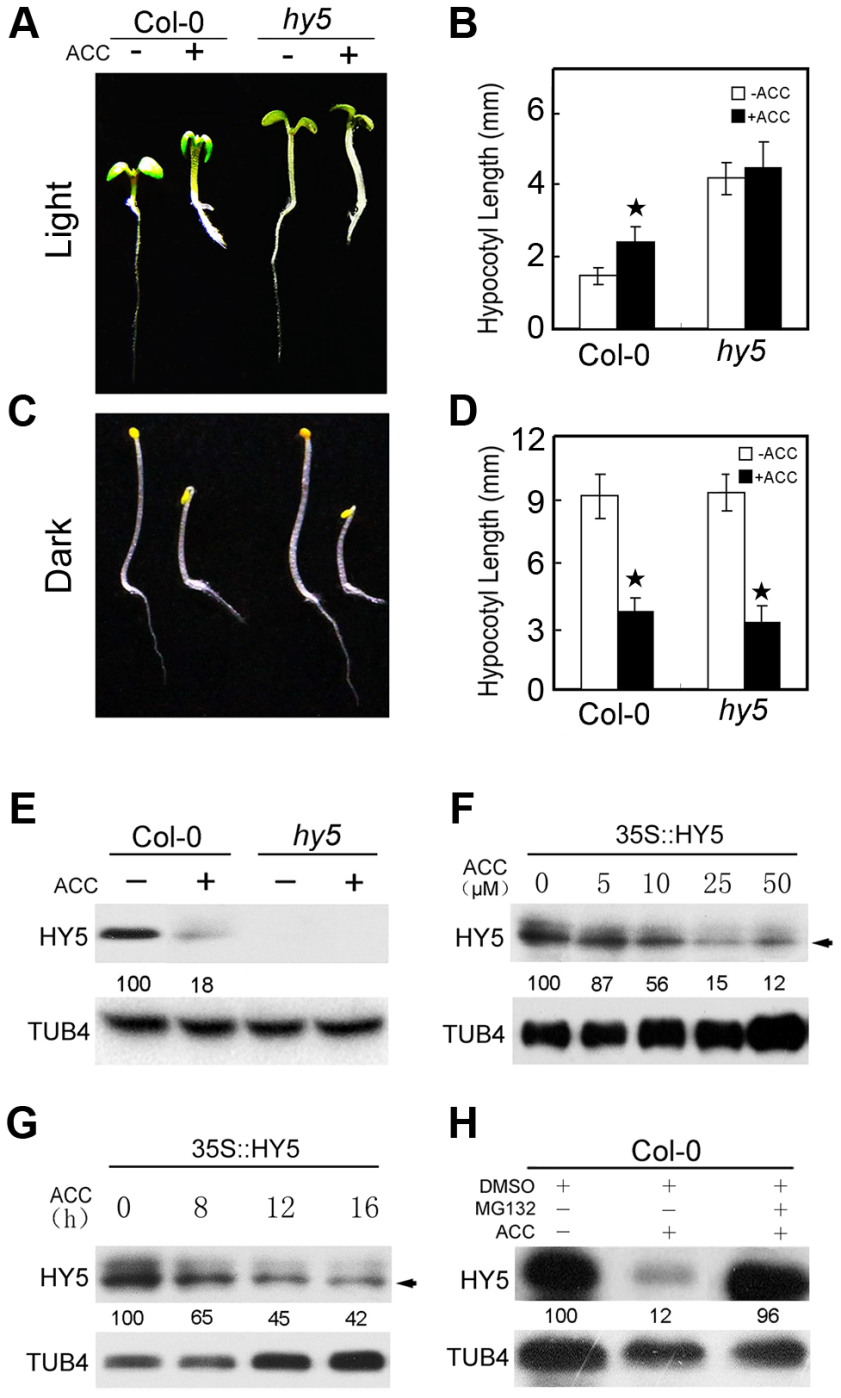
HY5 is required for ethylene-promoted hypocotyl growth in the light. (**A, C**) Morphological observations and (**B, D**) statistical analyses of hypocotyl length. Images were taken after 5 days of incubation in MS medium supplemented with or without 10 µM ACC. The data indicate the mean values plus the standard deviation (SD) from three independent experiments with approximately 30 seedlings. *P*-values (ACC treatment vs. non-treatment) were calculated by a two-tailed Student's *t*-test assuming equal variances (*P<0.05). (**E**) HY5 accumulation in Col-0 and *hy5*. Protein extracts were prepared from 5-day-old seedlings after treatment with or without 25 µM ACC for 15 h. (**F**) The effects of different ACC concentrations on HY5 stability in 35S::HY5 seedlings. (**G**) A time course of HY5 stability in response to ACC treatment in 35S::HY5 seedlings. Five-day-old seedlings were treated either with the indicated concentrations of ACC treatment for 16 h or with 25 µM ACC for the indicated times. The arrow in (**F**) and (**G**) indicates endogenous HY5, whereas the upper band corresponds to transgenic HY5. (**H**) Effect of the 26S proteolysis inhibitor MG132 on HY5 protein accumulation in Col-0 under normal growth conditions. Five-day-old seedlings were treated with 25 µM ACC plus 0.1% DMSO or 5 µM MG132 for 15 h. Immunoblotting was performed with anti-HY5 and -TUB4 antibodies. The TUB4 signals confirm equal protein loading. Numbers indicate the relative protein levels of HY5.

Ethylene confers opposing effects on hypocotyl growth that are dependent on the conditions under which plants are grown (light or dark). Ethylene and its precursor ACC can stimulate hypocotyl growth in the light, but inhibit hypocotyl elongation in etiolated seedlings. HY5 is an important positive regulator of photomorphogenesis; the *hy5* mutant displays a long hypocotyl when grown in the light, but a normal hypocotyl when grown in the dark [41]. A recent report revealed that HY5 binds to the promoters of cell elongation-related genes and recruits PKL/EPP1 through their physical interaction to regulate hypocotyl growth [42]. Interestingly, although treatment with 10 µM ACC diminished hypocotyl growth in dark-grown Col-0 and *hy5* plants, ACC did not significantly promote hypocotyl growth in light-grown *hy5* seedlings (Figure 1A–D). This result was confirmed in *hy5-215* (Col-0 background) and *hy5-ks50* (Ws background, Figure S1). In addition, the root of *hy5* mutants responded normally to ethylene because ACC inhibited the growth of root as the same of Col-0 in the light or in the dark (Figure 1A and 1C, Figure S1A). These results indicate that HY5 plays an essential role in ethylene-promoted hypocotyl growth. As a key factor in photomorphogenic development, HY5 represents a convergence point for light and multiple hormone signalling pathways [29]; combined with our results, these observations suggest that HY5 participates in ethylene-promoted hypocotyl elongation in the light but not in the dark.

To confirm the function of HY5 in regulating hypocotyl growth, we generated a hybrid of the ethylene overproducing mutant *eto2* [43] and transgenic plants constitutively expressing *HY5* (35S::HY5) [44]. As shown in Figure S2A and S2B, *eto2* displayed longer hypocotyls than Col-0 plants, while the hypocotyl length of *eto2*×35S::HY5 was intermediate between that of *eto2* and Col-0. The long hypocotyl caused by endogenous ethylene overproduction was diminished by the constitutive expression of *HY5* (Figure S2), indicating that HY5 negatively regulates ethylene-promoted hypocotyl growth in the light.

### Ethylene suppresses HY5 accumulation in a dose- and time-dependent manner

To determine how ethylene modulates HY5 accumulation, we first examined whether ethylene transcriptionally activates *HY5* expression. The expression levels of *HY5* and its homologue *HYH* were similar in Col-0, *eto*, *ein2*, and *ein3-1* under normal growth conditions (Figure S3), suggesting that ethylene does not transcriptionally regulate *HY5* expression. We next detected HY5 protein accumulation in Col-0 and *hy5* mutant plants. There was no band detected in *hy5* mutant while ACC greatly reduced HY5 protein accumulation in Col-0 (Figure 1E), suggesting that ethylene mediates an increase in HY5 instability at the post-transcriptional level to regulate hypocotyl growth.

To further understand how ethylene suppresses HY5 accumulation, we analysed the stability of HY5 in the transgenic line 35S::HY5. Because ACC induces hypocotyl growth in a dose-dependent manner [24], we first examined the induction of HY5 degradation by ethylene using different concentrations of ACC. Treatment with 5, 10, or 25 µM ACC for 16 h slightly, greatly, and significantly suppressed HY5 accumulation in 35S::HY5, respectively. When the applied ACC concentration increased, HY5 protein accumulation gradually decreased (Figure 1F). Furthermore, we observed that treatment with ACC for 8 h resulted in obvious degradation of HY5; when the treatment was increased to 16 h, the HY5 protein level was further reduced (Figure 1G), suggesting that ethylene significantly suppresses HY5 stability in a dose- and time-dependent manner. We also found that treatment with the 26S protein degradation inhibitor MG132 prevented the decrease in HY5 protein levels caused by ACC treatment (Figure 1H), suggesting that ethylene regulates HY5 stability via 26S proteasome-mediated degradation.

### HY5 acts downstream of EIN3 in ethylene-promoted hypocotyl elongation

**Figure 2:**
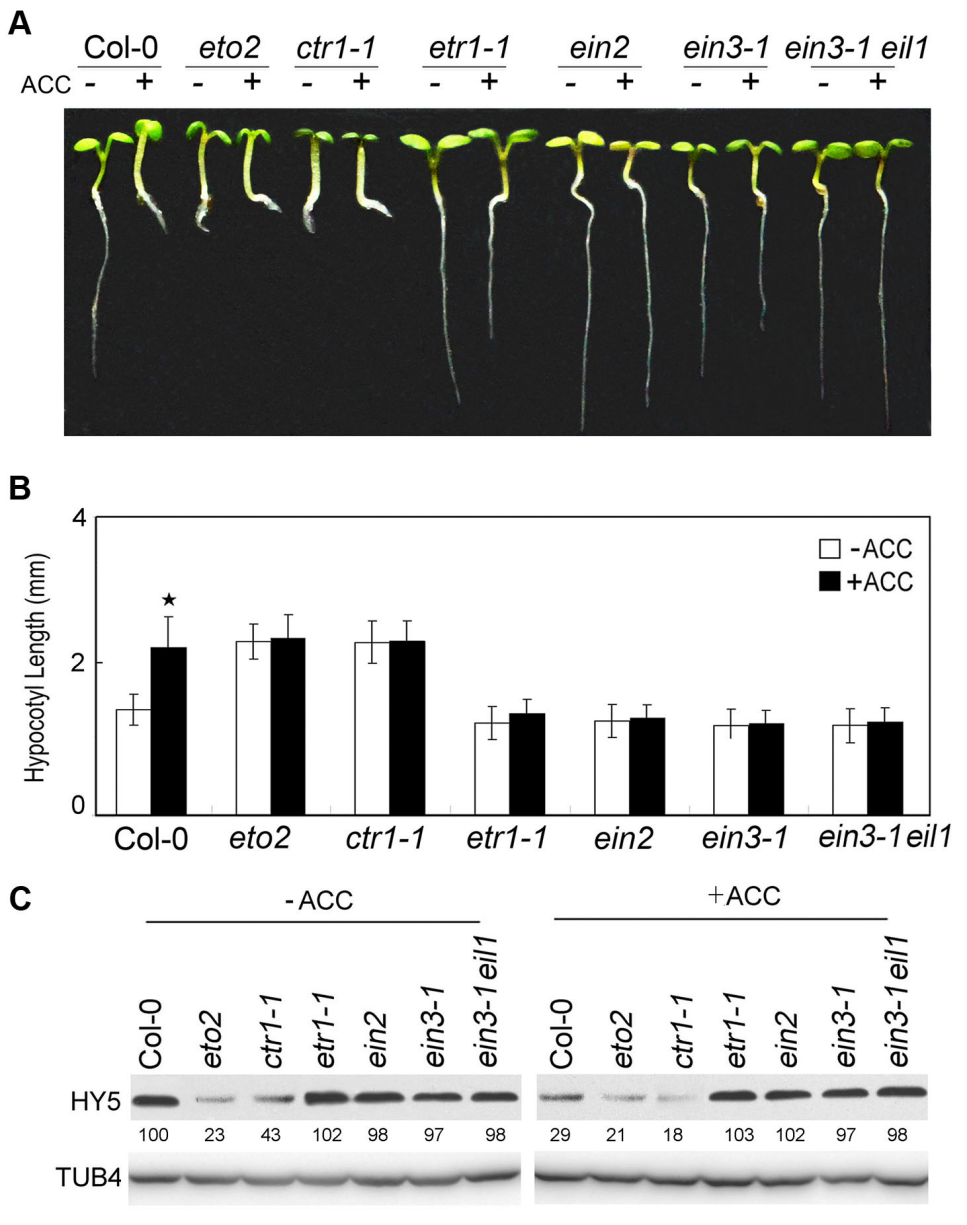
Ethylene signalling positively promotes hypocotyl growth and HY5 degradation. (**A**) Morphological observations and (**B**) statistical analyses of hypocotyl length after 5 days of incubation in MS medium supplemented with or without 10 µM ACC. The data indicate the mean values plus the SD from three independent experiments with approximately 30 seedlings. *P*-values (ACC treatment vs. non-treatment) were determined with a two-tailed Student's *t*-test assuming equal variances (*P<0.05). (**C**) HY5 accumulation after treatment with or without ACC treatment. Protein extracts were prepared from 5-day-old seedlings after treatment with or without 25 µM ACC for 15 h. Immunoblotting was performed with anti-HY5 and -TUB4 antibodies. The TUB4 signals indicate equal protein loading. Numbers indicate the relative protein levels of HY5.

To further study the mechanism of ethylene-promoted hypocotyl elongation in the light, we examined the hypocotyl lengths of ethylene signalling mutants. Similar to *eto2*, the constitutive ethylene response mutant *ctr1-1* displayed longer hypocotyls than Col-0 or the ethylene-insensitive mutants *etr1-1*, *ein2*, *ein3-1*, and *ein3-1 eil1*. Unlike Col-0, the hypocotyl lengths of *etr1-1*, *ein2*, *ein3-1*, and *ein3-1 eil1* did not increase following treatment with 10 µM ACC (Figure 2A and 2B). An analysis of HY5 expression in these mutants by Western blotting produced results that were consistent with those of our hypocotyl elongation assays (Figure 2C). Namely, HY5 accumulation was reduced in *eto2* and *ctr1-1* compared to Col-0 in the absence of ACC, and ACC treatment enhanced the decrease in HY5 protein, especially in *ctr1-1*. However, in *etr1-1*, *ein2*, *ein3-1*, and *ein3-1 eil1*, only slight differences from Col-0 were observed, and ACC did not promote HY5 degradation (Figure 2C). Consistent with the hypocotyl lengths shown in Figure 2A and 2B, these results indicate that HY5 acts downstream of ethylene signalling to regulate hypocotyl growth.

**Figure 3:**
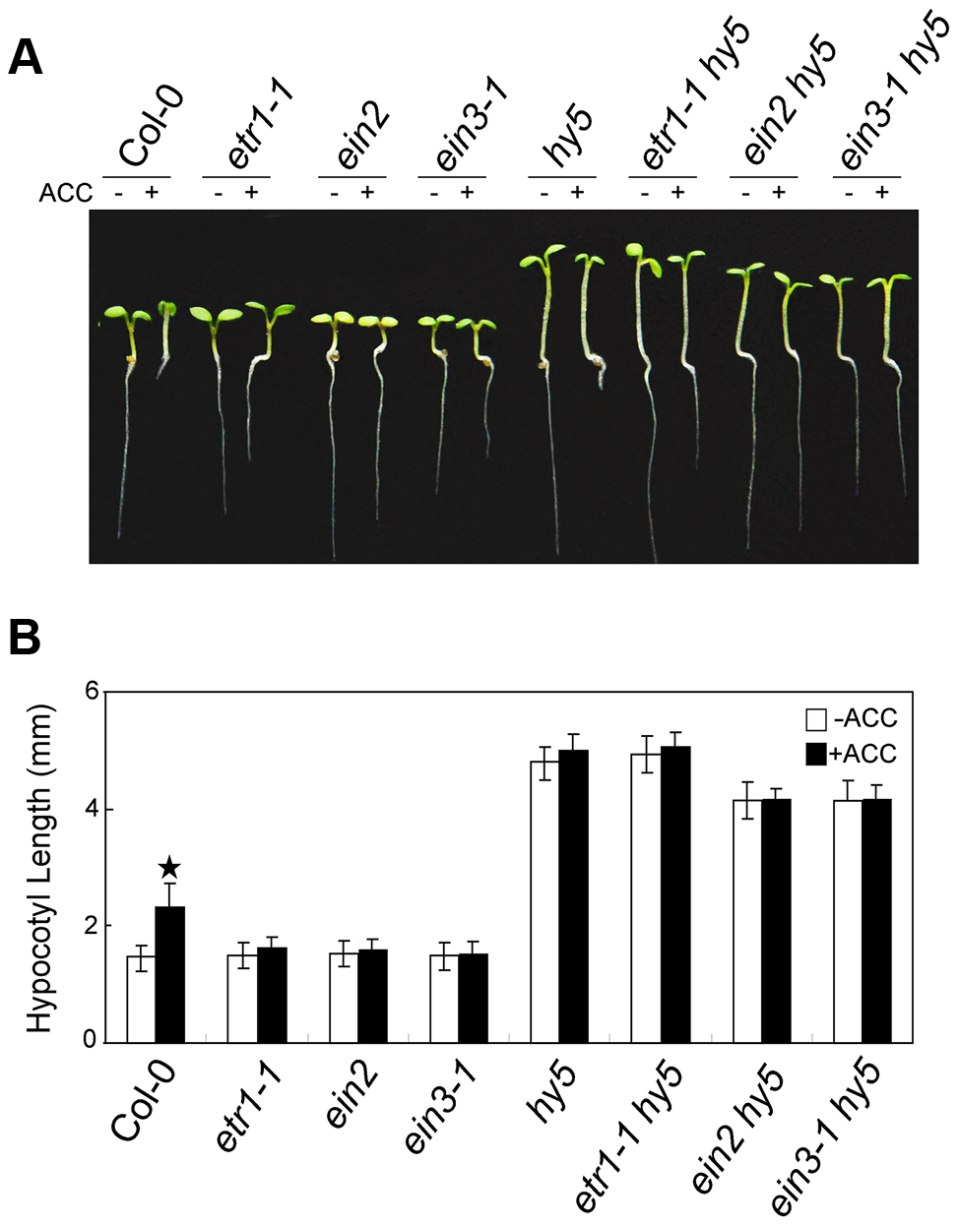
HY5 operates downstream of EIN3 to modulate ethylene-promoted hypocotyl growth. (**A**) Morphological observations and (**B**) statistical analyses of hypocotyl length after 5 days of incubation in MS medium supplemented with or without 10 µM ACC. The data indicate the mean values plus SD from three independent experiments with approximately 30 seedlings. *P*-values (ACC treatment vs. non-treatment) were determined with a two-tailed Student's *t*-test assuming equal variances (*P<0.05).

We next analysed the genetic relationship between HY5 and ethylene signalling. The hypocotyl lengths in the *etr1-1 hy5*, *ein2 hy5*, and *ein3-1 hy5* double mutants were obviously longer than that in Col-0 and similar to that in *hy5* (Figure 3A and 3B). Taken together with the finding that ACC did not promote the degradation of HY5 in *ein3-1* (Figure 2C), our data indicate that HY5 acts downstream of EIN3 to mediate ethylene-promoted hypocotyl growth.

### COP1 mediates ethylene-promoted HY5 degradation

**Figure 4:**
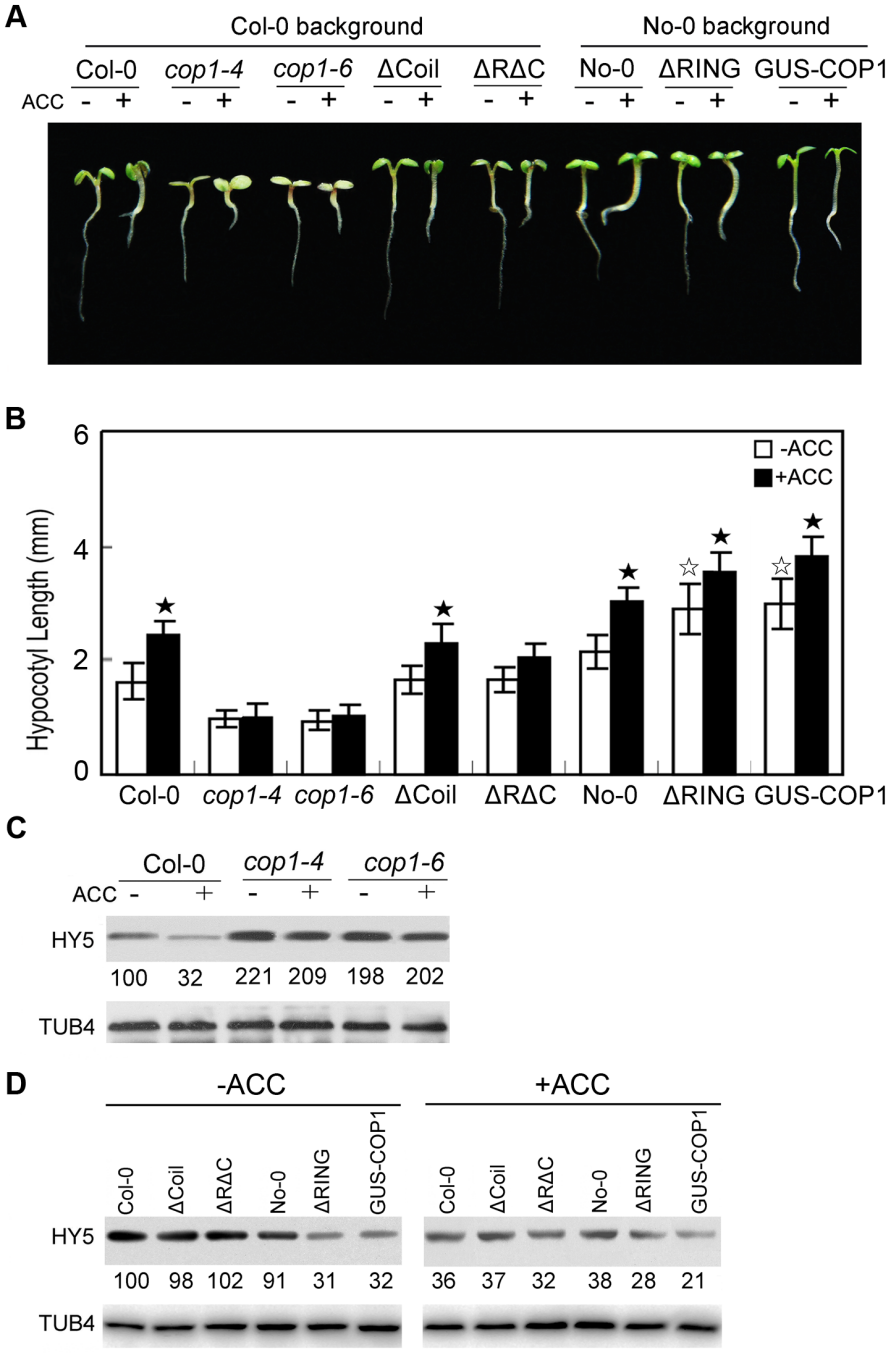
COP1 is required for ethylene-promoted hypocotyl growth. (**A**) Morphological observations and (**B**) statistical analyses of hypocotyl length after 5 days of incubation in MS medium supplemented with or without 10 µM ACC. The data indicate the mean values plus the SD from three independent experiments with approximately 30 seedlings. *P*-values (★: ACC treatment vs. non-treatment, ☆: transgenic lines vs. wild type) were determined with a two-tailed Student's *t*-test assuming equal variances (*P<0.05). (**C, D**) HY5 accumulation with or without ACC treatment. Protein extracts were prepared from 5-day-old seedlings after treatment with or without 25 µM ACC for 15 h. Immunoblotting was performed using anti-HY5 and -TUB4 antibodies. The TUB4 signals indicate equal protein loading. Numbers indicate the relative protein levels of HY5.

COP1 is an E3 ligase that negatively modulates HY5 activity via protein-protein interactions [33], [36]. COP1 has three structural domains that are critical for its molecular association with HY5: an N-terminal RING-finger domain followed by a predicted coiled-coil domain and C-terminal WD-40 repeats [33]. For the regulation of HY5 stability by ethylene through 26S proteasome-mediated degradation, we hypothesised that COP1 may also function in this process. Therefore, we measured the ethylene response in *Arabidopsis cop1* mutants (*cop1-4* and *cop1-6*, both in Col-0 background) and transgenic lines. It has previously evidenced that the *cop1-4* mutation alters the Gln-283 CAA codon to a UAA stop codon, resulting in a truncated COP1 protein containing only the N-terminal 282 amino acids, and the *cop1-6* mutation changes the splicing junction “AG” at the 3′ end of intron 4 to “GG”, generating a five-amino acid insertion in the COP1 protein [45]. Interestingly, those *cop1* mutants that reduced activity of COP1 displayed shorter hypocotyls than Col-0, and ACC failed to promote hypocotyl elongation in *cop1-4* or *cop1-6*, whereas the full-length *COP1* overexpressor (GUS-COP1) displayed longer hypocotyls than Col-0 (Figure 4A and 4B). These results indicate that COP1 positively regulates ethylene-promoted hypocotyl growth.

The RING-finger domain, coiled-coil domain, and WD-40 repeats of COP1 are essential for its interaction with HY5 [33], [46]. Accordingly, a transgenic line overexpressing *COP1* without the RING-finger domain (ΔRING, No-0 background) showed significantly longer hypocotyls than the wild type, whereas a transgenic line overexpressing *COP1* without the coiled-coil domain (ΔCoil, Col-0 background) displayed a similar hypocotyl length to the wild type in the absence of ACC under white light (Figure 4A and 4B). Distinctive to the *cop1* mutant, ACC significantly enhanced hypocotyl growth in the ΔRING and ΔCoil transgenic lines, similar to the effect of ACC on their corresponding controls (Figure 4A and 4B), suggesting the redundant function of the RING-finger and coiled-coil domains in hypocotyl growth. Furthermore, transgenic line overexpressing *COP1* without the RING-finger and coiled-coil domains (ΔRΔC, Col-0 background) did not show obvious hypocotyl elongation compared to Col-0 in the absence of ACC under white light, and ACC did not significantly improve hypocotyl growth in the ΔRΔC line (Figure 4A and 4B). Thus the above observations indicate that mutation of protein-protein interaction domains of COP1 alters ethylene responsive hypocotyl elongation.

To elucidate the mechanism through which ethylene suppresses HY5 accumulation, we analysed the HY5 protein levels in the *cop1* mutants and transgenic lines. As expected, HY5 accumulation was detected in *cop1-4* and *cop1-6*, while ACC-induced HY5 degradation was inhibited in *cop1-4* and *cop1-6* (Figure 4C), revealing the essential role of COP1 in ethylene-promoted hypocotyl growth and HY5 stability. Moreover, the abundance of HY5 in these transgenic lines was correlated with the observed extent of photomorphogenic development. When grown under the same white light conditions, GUS-COP1 and ΔRING had reduced while ΔCoil and ΔRΔC showed similar levels of HY5 protein relative to wild type, and ACC treatment enhanced the degradation of HY5 in these transgenic lines (Figure 4D), indicating that the ACC-induced suppression of HY5 is mediated by the E3 ligase COP1, dependent on the interaction of COP1 with HY5.

**Figure 5:**
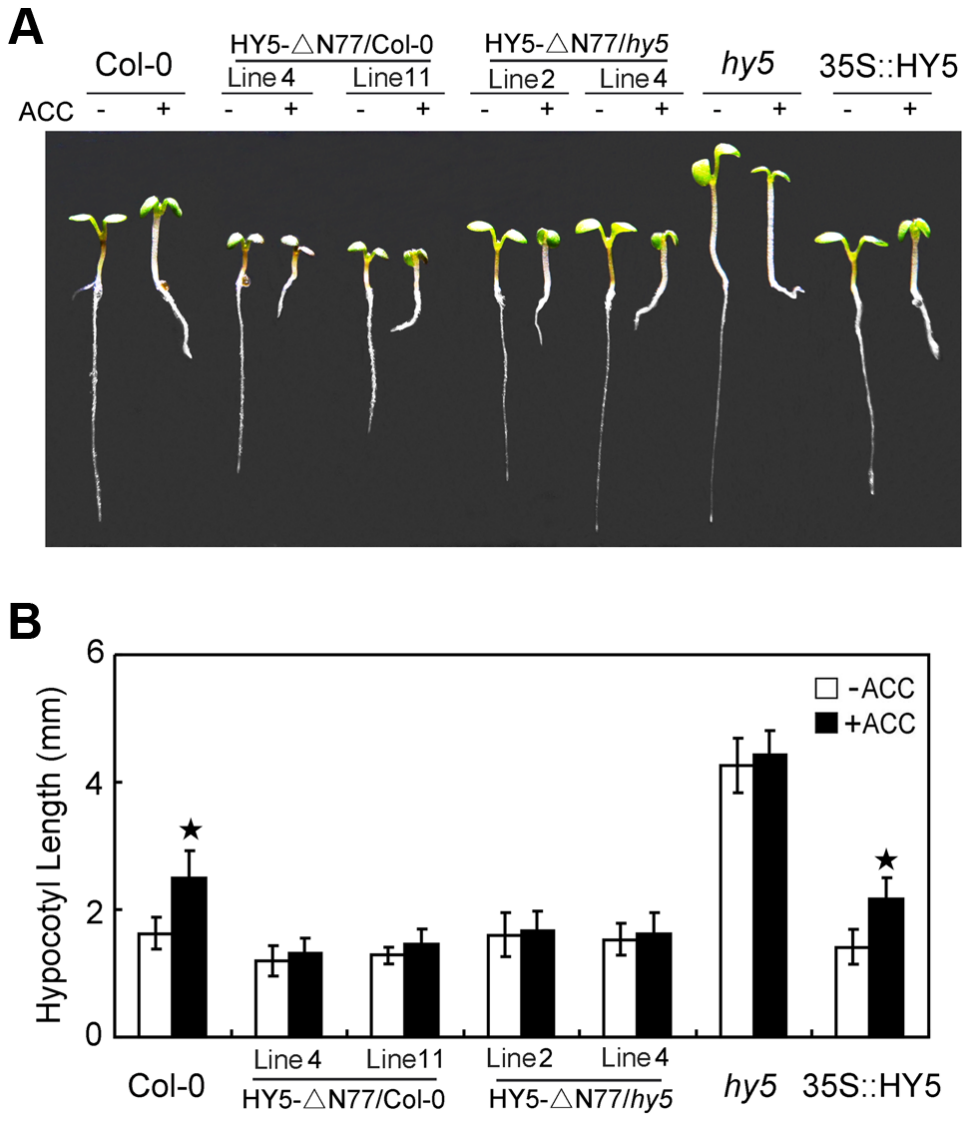
COP1-HY5 interaction is required for HY5-mediated hypocotyl growth. (**A**) Morphological observations and (**B**) statistical analyses of hypocotyl length in transgenic lines of HY5-ΔN77 driven by the CaMV35S promoter. The images were taken after 5 days of incubation in MS medium supplemented with or without 10 µM ACC. The data indicate the mean values plus the SD from three independent experiments with approximately 30 seedlings. *P*-values (ACC treatment vs. non-treatment) were determined with a two-tailed Student's *t*-test assuming equal variances (*P<0.05).

Because HY5 is not the unique targets of COP1 [47]–[49], then we further questioned whether the role of HY5 in ethylene-promoted growth is specifically targeted by the interaction with COP1. To address this query, we performed assays of ethylene responsiveness using transgenic lines of the deletion of N-terminal 77 amino acids of HY5 (HY5-ΔN77) in Col-0 or *hy5* background, which has been evidenced that this fragment is essential for the interaction of HY5 with COP1, and HY5-ΔN77 transgene is being driven by the CaMV35S promoter [33]. Transcriptional detections with quantitative real-time PCR (qPCR) showed that the expression of truncated *HY5* (deletion of the N-terminal 77 amino acids) or full-length *HY5* was overexpressed in individual transgenic lines (Figure S4). Further observations showed that the transgenic lines of HY5-ΔN77 displayed shortened-hypocotyl in Col-0 background, but the HY5-ΔN77 partially complemented the *hy5* long hypocotyl phenotype. More importantly, ACC did not significantly promote the elongation of hypocotyl in HY5-ΔN77/Col-0 and HY5-ΔN77/*hy5* (Figure 5). These results indicate that the COP1-HY5 interaction is required for the degradation of HY5 by ethylene, and subsequent hypocotyl growth.

**Figure 6:**
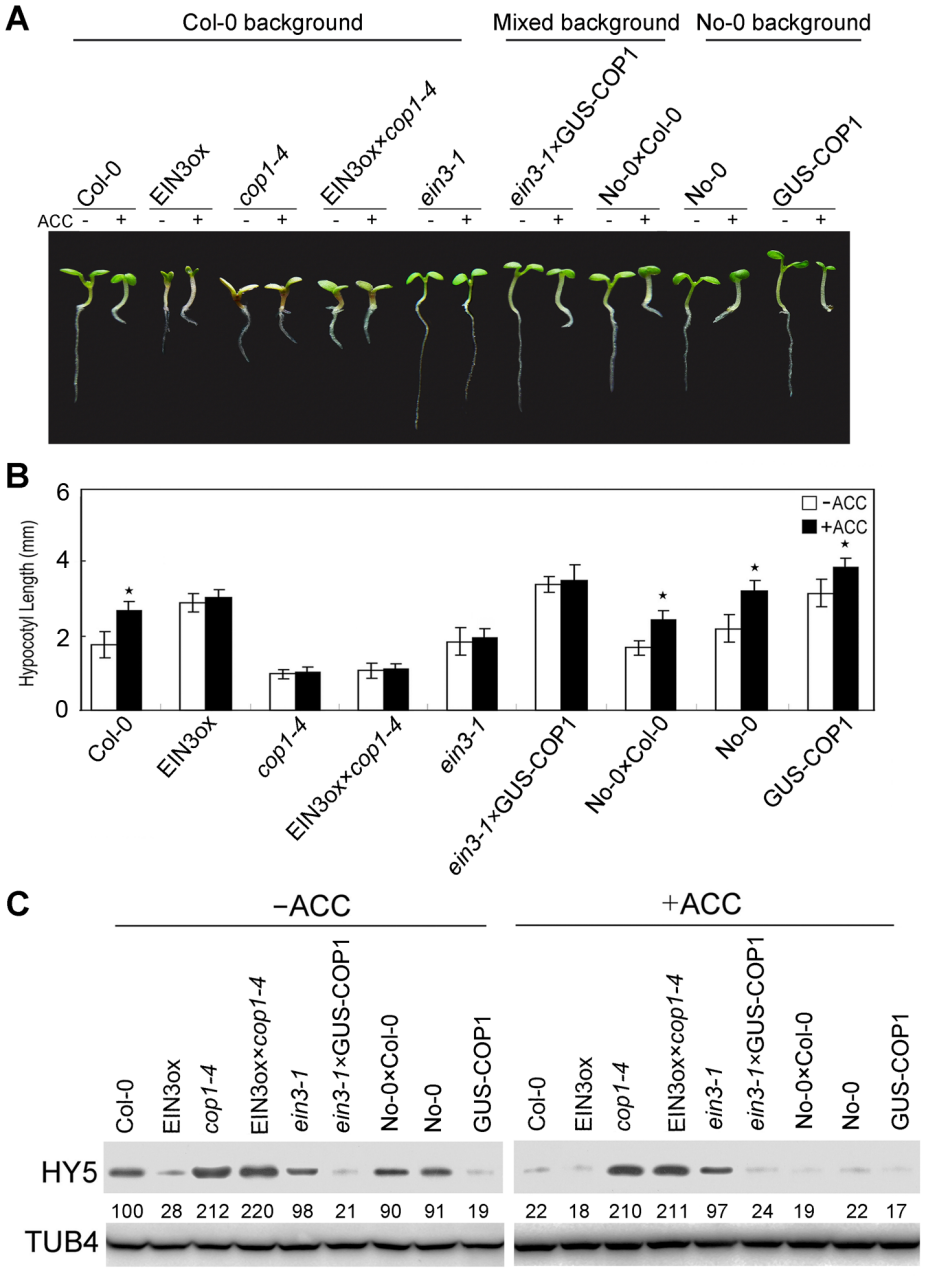
COP1 acts downstream of EIN3 to modulate ethylene-promoted hypocotyl growth. (**A**) Morphological observations and (**B**) statistical analyses of hypocotyl length after 5 days of incubation in MS medium supplemented with or without 10 µM ACC. The data indicate the mean values plus the SD from three independent experiments with approximately 30 seedlings. *P*-values (ACC treatment vs. non-treatment) were determined with a two-tailed Student's *t*-test assuming equal variances (*P<0.05). (**C**) HY5 accumulation with or without ACC treatment. Protein extracts were prepared from 5-day-old seedlings after treatment with or without 25 µM ACC for 15 h. Immunoblotting was performed with anti-HY5 and -TUB4 antibodies. The TUB4 signals indicate equal protein loading. Because the *ein3-1* and GUS-COP1 were produced in different backgrounds, an F1 hybrid of No-0×Col-0 was used as the control. The numbers below the gel indicate the protein levels of HY5.

We then generated *eto2 cop1-4* and *ctr1 cop1-4* double mutants to determine whether ethylene-mediated hypocotyl elongation is COP1-dependent. Both of the mutants displayed hypocotyl lengths that were similar to those of *cop1-4* and were not affected by ACC treatment (Figure S5). To investigate the relationship between COP1 and ethylene signalling, we evaluated the hybrids EIN3ox×*cop1-4* and *ein3-1*×GUS-COP1. Both EIN3ox and GUS-COP1 displayed longer hypocotyls than the controls, indicating that EIN3 and COP1 positively regulate ethylene-promoted hypocotyl growth. However, EIN3ox×*cop1-4*, which lacked COP1, displayed a short hypocotyl phenotype that was similar to that of *cop1-4*, and *ein3-1*×GUS-COP1, which lacked EIN3, showed the same long hypocotyl phenotype as GUS-COP1 (Figure 6A and 6B), indicating that COP1 might be genetically downstream of EIN3. This conclusion was supported by Western blotting analyses: as shown in Figure 6C, twice as much HY5 protein accumulated in EIN3ox×*cop1-4* as in *cop1-4*, and this accumulation was not affected by ACC treatment. Correspondingly, the HY5 level in *ein3-1*×GUS-COP1 was dramatically reduced compared to that in the controls (Figure 6C), consistent with the function of HY5, COP1 likely acts downstream of EIN3 in ethylene-promoted hypocotyl elongation. Thus, our results demonstrate that the ethylene-enhanced degradation of HY5 is mediated by COP1 and that the COP1-HY5 complex acts downstream of EIN3 to mediate ethylene-promoted hypocotyl growth.

To further confirm the ethylene responsiveness of the physical interaction between COP1 and HY5, we examined the expression of the ethylene-regulated genes *ERF1* [16], *ESE1* [21], and *CHIB* [50] in *hy5*. Our data show that the expression of *ERF1*, *ESE1*, and *CHIB* was inhibited in *cop1-4*, but increased in *hy5* (Figure S6), indicating the participation of COP1-HY5 not only in ethylene-promoted hypocotyl growth, but also in ethylene-regulated gene expression.

Interestingly, we did not observe an obvious difference in root length between Col-0 and the ethylene signalling mutants in the absence of ACC. In particular, root growth in the mutants *eto*2 and *ctr1-1* was constitutively inhibited. However, ACC significantly diminished root growth in Col-0, but not in *ein2* or the *ein3 eil1* double mutant, and it partially inhibited root growth in *etr1-1* and *ein3-1* (Figure S7A), demonstrating the essential role of ethylene in the regulation of root growth. However, the root length in the *hy5* or *cop1* mutants and the transgenic lines expressing truncated versions of *COP1* was reduced by treatment with ACC (Figure S7B), indicating that the COP-HY5 complex mainly takes part in ethylene-regulated hypocotyl growth rather than root growth.

### Ethylene enhances COP1 nuclear localisation

**Figure 7:**
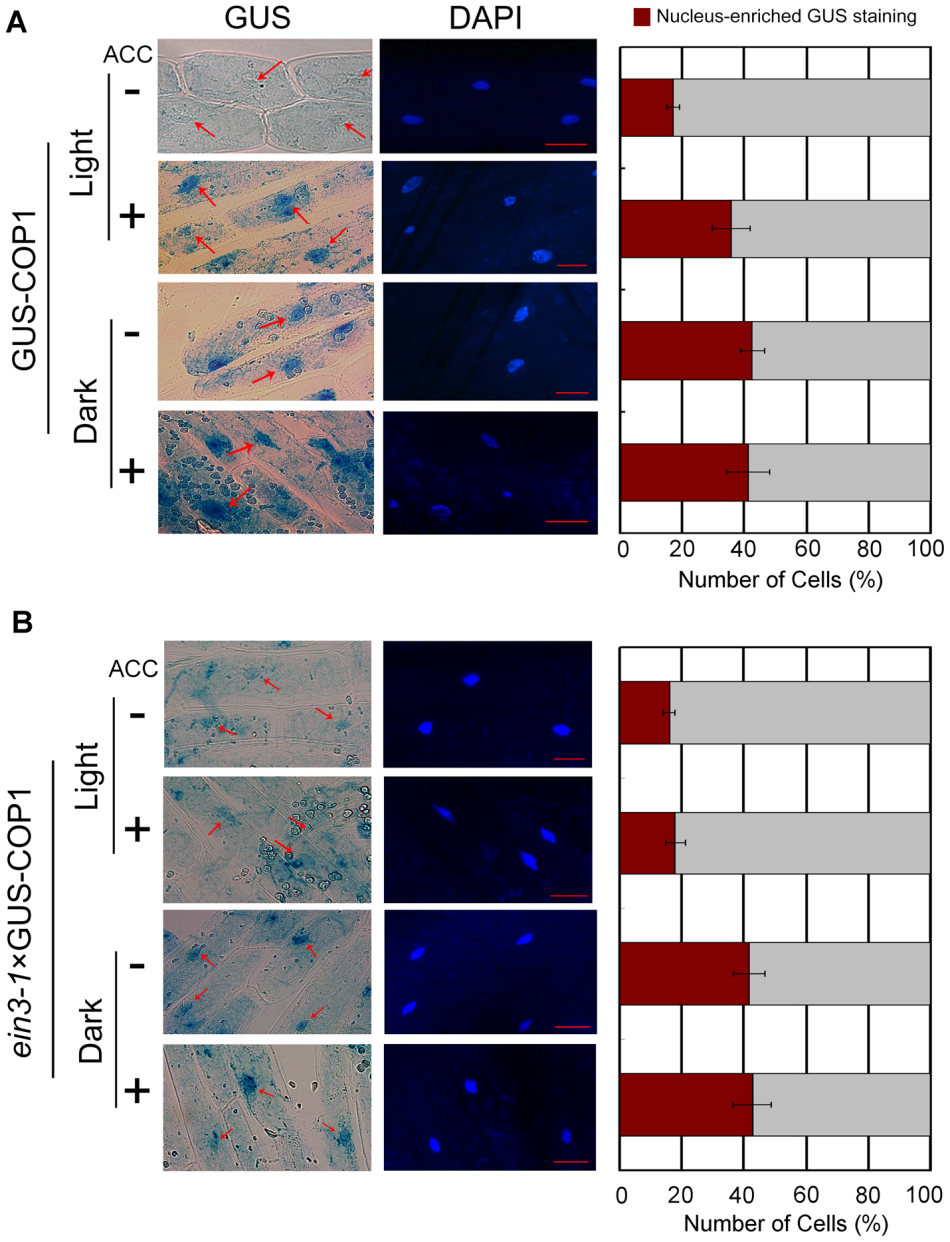
The ethylene precursor ACC improves COP1 nucleus-enriched localisation in the light. Images (left panel) and statistical summaries (right panel) of GUS-COP1 localisation in the hypocotyl in Col-0 (**A**) and *ein3-1* (**B**) under different growth conditions. The degree of nuclear enrichment of GUS staining is shown as the percentage of cells with nucleus-enriched GUS relative to the total number of GUS-stained hypocotyl cells. At least 100 cells were counted for each sample. GUS-COP1 transgenic seedlings were first grown on MS for 5 days and then grown in the dark (labelled “Dark”) or under continuous white light (50 µmol/m^2^s; labelled “Light”) for another 24 h with or without 25 µM ACC. The cell nuclei were stained with 0.5 µg/mL DAPI. Scale bar: 5 µm.

COP1 activity has been correlated with its movement between the cytoplasm and nucleus [35]. COP1 is excluded from the nucleus in the light, whereas in the dark or shade it accumulates in the nucleus and directs its targets, including HY5, to the proteasomal degradation machinery [36], [47]–[49], [51], [52]. This nucleocytoplasmic partitioning of COP1 is required to promote HY5 protein accumulation [53], [54]. To determine whether ethylene influences COP1 localisation to the nucleus, we quantified the nuclear and cytoplasmic localisation of COP1 in a transgenic line that constitutively expresses a GUS-COP1 fusion (GUS-COP1). GUS was mainly localised to the nucleus in the dark, but it translocated to the cytosol upon light irradiation in the absence of ACC (Figure 7A and 7B), consistent with previous data [35]. Importantly, ACC treatment did not induce obvious changes in GUS localisation in the dark, whereas under continuous white light for 24 h, ACC treatment enhanced the nuclear localisation of GUS-COP1 (Figure 7A), suggesting that ACC affects the distribution of COP1 and hypocotyl growth in the light rather than in the dark. Further, we used 4-day-old seedlings grown in long days (16/8 h), and then the plants were shifted to continuous white light for 36–48 h to conduct experiments. The level of GUS staining in the nucleus did not change following treatment with ACC under continuous white light (Figure S8). Moreover, under an 8-h/16-h dark/light cycle approximately 18% of the hypocotyl cells exhibited nucleus-enriched GUS staining in the absence of ACC; however, after addition of ACC under the same growth conditions, the proportion of cells with nucleus-enriched GUS staining increased to 58% (Figure S9A–D). These results indicate that ACC reversed the localisation of COP1 in the light and further enhanced HY5 degradation.

Because COP1 is a downstream component of EIN3, we examined whether ethylene modulates COP1 localisation via the action of EIN3. To address this issue, we constructed a hybrid of GUS-COP transgenic line using the *ein3-1* mutant to detect GUS-COP1 shuttling between the cytoplasm and nucleus and grew the hybrid under continuous white light for 24 h. Our results indicate that the loss of function of *EIN3* in the *ein3*×GUS-COP1 hybrid disabled the shuttling of COP1 to the nucleus promoted by ACC. In comparison, the shuttling of COP1 in *ein3*×GUS-COP1 was controlled by darkness (Figure 7B). These results demonstrate that ethylene modulates COP1 localisation via the action of EIN3.

**Figure 8:**
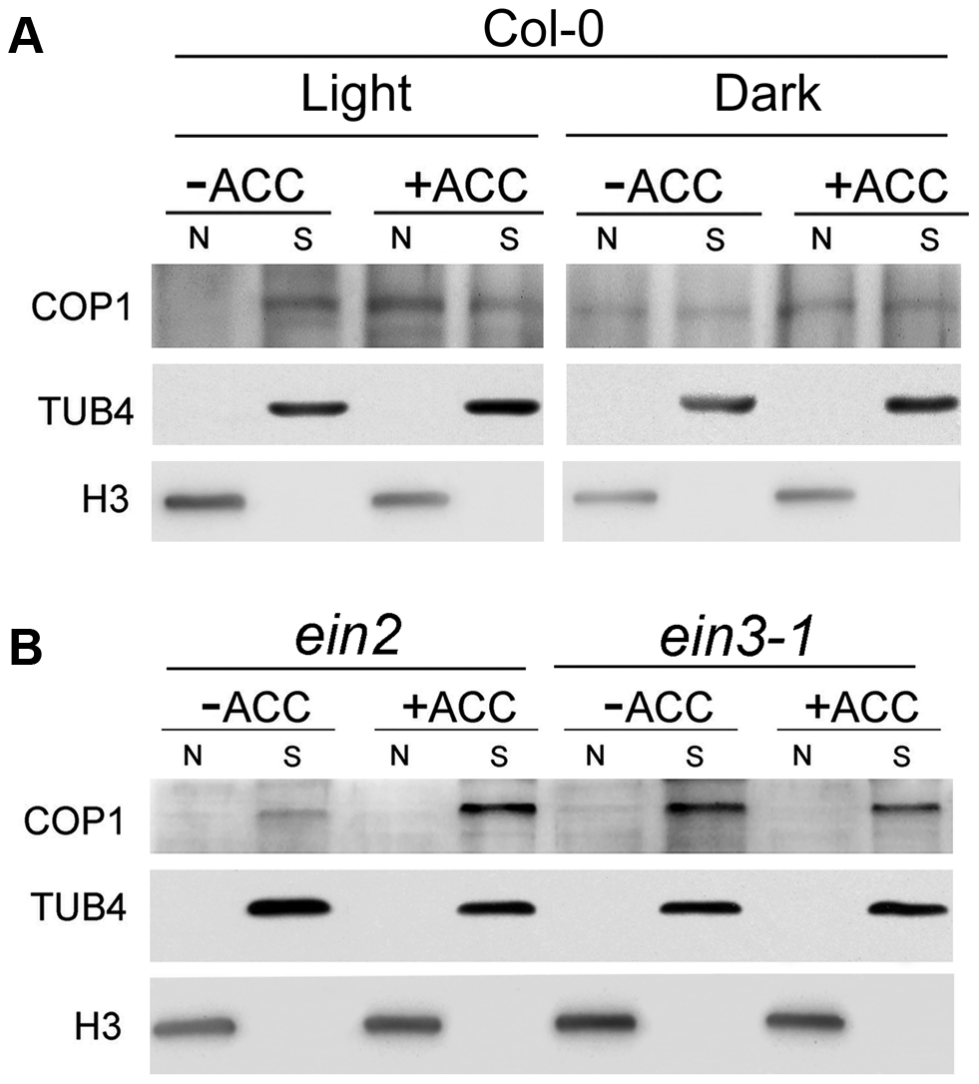
Biochemical detection of COP1 in the cytoplasm and nucleus. Seedlings of five-day-old (**A**) Col-0 and (**B**) *ein2* and *ein3-1 Arabidopsis* plants grown under long-day conditions (16-h light/8-h dark) were treated with 50 µM CHX and 5 µM MG132, supplemented with or without 25 µM ACC for 15 h, and then the nuclear proteins were extracted as described in the Materials and methods (N: nuclear protein, S: soluble fraction, cytoplasmic protein). Immunoblotting was performed using anti-COP1, -histone 3 (H3), and -TUB4 antibodies. The TUB4 and H3 signals confirm equal protein loading. All of the seedlings were illuminated with white light (50 µmol/m^2^s).

To directly demonstrate that ACC promotes the shuttling of COP1 between the nucleus and cytoplasm, we performed cell fractionation experiments using 5-day-old Col-0 plants grown under an 8-h/16-h dark/light cycle. To exclude the influence of *de novo* protein production and degradation during the process, we supplemented with cycloheximide (CHX) and MG132 with or without ACC in the light for 15 h. Our results showed that COP1 was mainly localized in the cytoplasm in the light without ACC treatment, while it shuttled to the nucleus in the presence of ACC (Figure 8A), evidencing that ACC enriches the nuclear distribution of COP1 in the light. Therefore our data suggest that ACC promotes COP1 translocation to the nucleus, resulting in the subsequent degradation of HY5 via 26S proteolysis. This experiment was also performed using *ein2* and *ein3-1* plants. In accordance with the localisation of GUS-COP1 in the *ein3-1* background, COP1 was only weakly detected in the nucleus with or without ACC (Figure 8B), demonstrating that ethylene-regulated COP1 shuttling occurs downstream of EIN3.

## Discussion

To understand the interactions between environmental cues and phytohormones, it is critical to elucidate the coordination of ethylene and light signalling during seedling hypocotyl growth. A recent study showed that EIN3 transcriptionally activates the expression of PIF3 and ERF1 to integrate these pathways [39], revealing the transcriptional regulation of a light regulator by ethylene. The bZIP transcriptional regulator HY5 has been shown to be a key factor in hypocotyl growth through its interaction with COP1 as a photomorphogenic repressor [33], [44]. In the present report, we demonstrated that the light regulators COP1 and HY5 are essential for ethylene-promoted hypocotyl growth in the light but not in the dark, and that these proteins act downstream of the ethylene signalling pathway component EIN3. Importantly, the ethylene-dependent activity of EIN3 enhanced the shuttling of COP1 between the nucleus and cytoplasm to promote HY5 degradation. Therefore, to our knowledge, this study presents the first evidence that the COP1-HY5 complex integrates ethylene-promoted hypocotyl growth in the light, increasing our understanding of the mechanism by which light suppresses (while ethylene promotes) hypocotyl elongation in the light.

Ethylene suppresses hypocotyl growth through the ethylene-induced gene *ERF1* whereas it promotes hypocotyl elongation downstream of EIN3 via *PIF3* in the light [39]. This is consistent with the finding that PIF3 and HY5 independently regulate PHYB-mediated hypocotyl elongation inhibition [55]. Moreover, regulation of the biosynthesis, transport, and distribution of IAA by ethylene was observed during light-mediated hypocotyl growth, further confirming the transcriptional regulation of hypocotyl growth by ethylene [29], [40]. In addition, increasing evidence indicates that the components of the ethylene signalling pathway play critical roles in plant growth and development [56]–[58]. In the present investigation, we demonstrated that ethylene-promoted hypocotyl growth in the light, but not in the dark, is mediated by the photomorphogenic factor HY5. The hypothesis that HY5 functions downstream of the ethylene signalling pathway is further supported by biochemical assays demonstrating that, under normal growth conditions, HY5 protein levels were not altered in loss-of-function ethylene signalling mutants but were significantly depressed in the *ctr1-1* background. Moreover, the addition of ACC greatly reduced HY5 accumulation in *eto2*, *ctr1-1*, and transgenic EIN3ox plants but not in *etr1-1*, *ein2*, *ein3*, and *ein3-1 eil1* plants. Therefore, the results of our genetic and phenotypic analyses and biochemical assays suggest that HY5 represents a novel negative regulator that functions downstream of the ethylene signalling component EIN3 and participates in ethylene-promoted hypocotyl growth.

The direct interaction between COP1 and CSN1 reportedly stimulates the nuclear localisation of COP1 [59], and the plant CSN-interacting F-box protein COP9 INTERACTING F-BOX KELCH 1 contributes to the control of hypocotyl elongation [60]. Indeed, several components of the photomorphogenic response, including CSN3, CSN6A, and CSN6B, interact with EIN2. It has been reported that EIN2 probably functions to direct the activity of the CSN through ENHANCED ETHYLENE RESPONSE 5, which is part of the ethylene response pathway [61], suggesting that photomorphogenic factors are involved in ethylene signalling. Importantly, we found that the EIN3-dependent nuclear localisation of COP1 could be enhanced by ethylene in the light but not in the dark, suggesting that ethylene affects the shuttling of COP1 to the nucleus. The *cop1* mutant has been shown to display a photomorphogenic response in the dark but a short hypocotyl in the light [35]. In the present study, exogenous ACC and endogenous ethylene stimulated hypocotyl growth, but this stimulation was not observed in *cop* mutant plants. These results suggest that ethylene-mediated hypocotyl growth is COP1-dependent, consistent with the finding that COP1 is involved in the ethylene-promoted photomorphogenic response [19], [40]. We found that *cop1* mutants have shorter hypocotyls, while *eto2* and *ctr1* display longer hypocotyls, in the light. The short hypocotyl lengths of the *eto2 cop1-4* and *ctr1 cop1-4* double mutants, however, were similar to that of the *cop1-4* mutant, and this phenotype was unaffected by the addition of ACC. Furthermore, genetic analyses and biochemical assays showed that the hypocotyl length and HY5 stability in the hybrids EIN3ox×*cop1-4* and *ein3-1*×GUS-COP1 were consistent with the function of COP1, indicating that COP1 acts downstream of the ethylene signalling component EIN3, similar to the manner in which HY5 is regulated by EIN3 during hypocotyl growth.

Interestingly, we also found that ACC enhanced the movement of COP1 to the nucleus in root cells upon light illumination (Figure S9E and S9F), suggesting the general regulation of the movement of COP1 in the light by ethylene. However, root elongation in *hy5*, *cop1*, and Col-0 seedlings was not significantly affected by ACC treatment, suggesting that the effect of ACC/ethylene on root growth is independent of COP1-HY5. Moreover, the length of the root in the ethylene signaling mutants was similar to that in Col-0 in the absence of ACC treatment. After ACC treatment, root growth was completely abolished in Col-0, but it was similar in terms of length in *ein2* and *ein3-1 eil1* with the control, implying that ethylene is essential for root growth. Reflecting the redundancy of EIN3 and EIL1, root growth in *ein3-1* and the gain-of-function mutant *etr1-1* was partially inhibited, compared to the corresponding controls. This observation suggests that the regulatory role of ethylene in hypocotyl growth is different from that in root elongation, further supporting the regulation of HY5 in hypocotyl growth rather than in root growth. Thus, the involvement of HY5 in the ethylene response might be an organ-specific effect.

Importantly, the data from our phenotypic and biochemical analyses indicate that a loss of function of *CTR1* constitutively improves hypocotyl growth by decreasing the level of HY5, while the hypocotyl length in *ctr1* is similar to that in ACC-treated Col-0, but shorter than that in *hy5*, suggesting that other factors coordinate with ethylene to affect HY5 protein levels. This hypothesis is supported by the observation that ACC moderately induces but does not significantly improve growth of the hypocotyl in the *hy5* mutant, consistent with previous observations [40], indicating that ethylene coordinates with other phytohormones, including auxin, to affect hypocotyl growth [62], [63].

The finding that ethylene promotes hypocotyl elongation in the light by increasing nuclear-localised COP1, resulting in a decreased level of HY5, combined with the observation that the effect of ethylene on HY5 protein levels was abolished in *etr1-1*, *ein2*, *ein3-1*, and *ein3/eil1*, indicates that the effect of ethylene on hypocotyl growth requires transcriptional regulation by EIN3. This hypothesis is supported by the following observations. *EIN3* overexpression decreased the HY5 protein level in a Col-0 background but not in a *cop1-4* background, whereas *COP1* overexpression reduced the HY5 level irrespective of EIN3. Moreover, the loss of function of EIN3 abolished the movement of COP1 in the light, suggesting that EIN3 is required for the COP1-mediated decrease in HY5. Therefore, based on the present data and previous reports showing that ethylene promotes EIN3/EIL1 accumulation whereas high EIN3/EIL1 levels are detrimental to plant growth and development [14], [15], we propose that COP1-HY5 functions as a regulatory module in ethylene-promoted hypocotyl growth. In the absence of ethylene, COP1 accumulates mainly in the cytoplasm in the light, releasing the suppression of COP1 on HY5. In the presence of ethylene, this effect is reversed, even though in the light the nucleus is enriched with COP1, resulting in the degradation of HY5. Ethylene production is negatively regulated by HY5 [64], [65]; decreases in HY5 accumulation enhance the synthesis of ethylene. Thus, in the present study, we found that the COP1-HY5 complex acts as an integrator downstream of EIN3 to fine-tune the regulation of hypocotyl growth by light and ethylene. Although previous data [19], [39], [40] and our findings suggest that cotyledon greening and hypocotyl growth are COP1-related, multiple factors downstream of COP1 may participate in these growth processes. Additional genetic and biochemical tests should be performed to better understand this integration.

## Materials and Methods

### Plant materials and growth conditions

All *Arabidopsis thaliana* mutants and transgenic lines were generated in the Columbia (Col-0) background with the exception of overexpression line of GUS-COP1, overexpressing *COP1* without the RING-finger domain (ΔRING, Nossen, No-0 background) and *hy5-ks50* (Wassilewskija, Ws background). Homozygous *eto1-1* (CS3072), *eto2* (CS8059), *eto3* (CS8060), *etr1-1* (CS237), *ctr1-1* (CS8057), *ein2* (CS8844), *ein3-1* (CS8052), and *hy5* (SALK_096651) lines were obtained from the *Arabidopsis* Biological Resource Center (Columbus, OH). The sequence data from this article can be found in the *Arabidopsis* Genome Initiative or GenBank/EMBL databases under the following accession numbers: *COP1* (At2g32950), *HY5* (At5g11260), *HYH* (At3g17609), *ETR1* (Ag1g66340), *CTR1* (At5g03730), *EIN2* (At5g03280), *EIN3* (At3g20770), *EIL1* (At2g27050), *ERF1* (AT3G23240), *ESE1* (AT3G23220), and *CHIB* (AT3G12500). The mutants or transgenic plants *hy5-215* and *hy5-ks50* [41]; *cop1-4* and *cop1-6* [45]; *ein3-1 eil1*, EIN3ox, and EIN3ox×c*op1-4* [19]; 35S::HY5 [44]; ΔRING, ΔCoil, and ΔRΔC [46]; HY5-ΔN77/Col-0 and HY5-ΔN77/*hy5* driven by the CaMV35S promoter [33] were generated previously. Seeds were surface-sterilised and sown on Murashige and Skoog (MS) medium containing 3% sucrose and 0.5% Phytagel. The plates were chilled at 4°C in the dark for 3 days and then moved to 22°C under a 16-h white light (50 µmol/m^2^s)/8-h dark cycle. All of the chemicals used were obtained from Sigma-Aldrich (St. Louis, MO).

### RNA extraction, reverse transcription, and quantitative real-time PCR (qPCR)

Total RNA was extracted from 5-day-old seedlings using TRIzol reagent (Invitrogen, Carlsbad, CA) and treated with RNase free-DNase I (Promega, Madison, WI) before the latter procedures were performed. Five micrograms of total RNA were reverse-transcribed to cDNA with M-MLV reverse transcriptase (Promega) according to the manufacturer's instructions. Gene expression was measured by qPCR analysis (SYBR Premix; Takara Bio Inc., Shiga, Japan). All amplification reactions were performed in 96-well optical reaction plates with 45 cycles of denaturation for 15 s at 95°C, annealing for 20 s at 56°C, and extension for 45 s at 72°C. The expression levels were normalised to that of TUB4. The primers used for qPCR are listed in Table S1. Each qPCR was repeated independently three times with the same expression pattern.

### Hypocotyl measurements

For the measurement of hypocotyl length, surface-sterilised seeds were deposited on plates containing MS medium with or without ACC. The plant materials and growth conditions were described in the previous section. At least 50 seedlings were measured from digital images of 5-day-old seedlings using ImageJ software.

### Western blot analysis

Proteins were extracted from approximately 50 seedlings treated with or without ACC in 100 µL of extraction buffer (50 mM Tris, pH 7.5, 150 mM NaCl, 1 mM EDTA, 10 mM NaF, 25 mM β-glycerophosphate, 2 mM sodium orthovanadate, 10% glycerol, 0.1% Tween 20, and 1 mM PMSF). After centrifugation, 10 µg of the supernatant was separated via SDS-PAGE and Western blotting was performed as described previously [36] using anti-HY5 antibodies (1∶500). TUB4 (1∶5,000) was used as a loading control.

The relative protein levels of HY5 were calculated using Image Gauge V3.12 (Fujifilm, Tokyo, Japan). The values were normalised to 100 in Col-0 without ACC treatment based on the TUB4 signal as an internal loading control.

### Genetic manipulation

35S::HY5 and *eto2* plants were crossed, and putative homozygous F2 progeny were screened for kanamycin resistance during seed germination. Kanamycin-resistant homozygous *eto2* seedlings were identified as described previously [66]. Non-segregating lines of the progeny of the *eto2* homozygous kanamycin-resistant plants were screened to identify homozygous hybrids of *eto2*×35S::HY5.

The double mutants *ein2 hy5*, *ein3-1 hy5*, and *etr1-1 hy5* were generated by crossing the recessive ethylene mutant *ein2*, *ein3-1*, or *etr1-1* with *hy5*. The F2 progeny of the crosses were grown in the dark for 4 days with 10 µM ACC to isolate ethylene-insensitive individuals that were homozygous for *ein2*, *ein3-1*, or *etr1-1*. The isolated individuals were subsequently screened for the presence of long hypocotyls, and the homozygous *hy5* mutations were confirmed by PCR-based genotyping.

The double mutants *eto2 cop1-4* and *ctr1-1 cop1-4* were generated by crossing the ethylene mutant *eto2*, or *ctr1-1* with *cop1-4*. The F2 progeny from the crosses were grown in the dark for 4 days to isolate homozygous *cop1-4* lines by the phenotype. Individuals were subsequently screened by PCR-based genotyping, and the PCR products were digested with *Ava*I (New England Biolabs, Ipswich, MA) for *eto2* or *Tsp*509I (New England Biolabs) for *ctr1-1* to identify homozygous double mutant lines. The primer information is summarised in Table S1.

*ein3-1* and GUS-COP1 plants were crossed, and the resulting putative homozygous plants (F2 progeny) were screened for kanamycin resistance during seed germination. After the identification of non-segregating progeny of the *ein3-1* homozygous plants, seedlings were screened to identify homozygous hybrids of *ein3-1*×GUS-COP1.

### Subcellular localisation of GUS-COP1

Transgenic GUS-COP1 seeds were germinated and grown on MS medium for 5 days. After treatment with or without 25 µM ACC for 24 h in the dark, continuous light, or light-dark (16 h/8 h) conditions, the seedlings were fixed with cold acetone and then soaked in GUS-DAPI buffer (0.5 µg/mL DAPI, 0.5 mg/mL 5-bromo-4-chloro-3-indolyl-β-D-glucuronic acid, 5 mM ferricyanide and ferrocyanide, 10 mM EDTA, and 0.1% [v/v] Tween 20 in 100 mM sodium phosphate, pH 7.0) and left in the dark at 37°C overnight. The tissues were cleared in 70% (v/v) ethanol and mounted on glass slides. To quantify the nuclear-cytoplasmic localisation of GUS-COP1, we calculated the percentages of cells that exhibited nucleus-enriched GUS staining. The cells in which GUS-COP1 staining was stronger in the nucleus than in the cytoplasm were scored as ‘nuclear,’ and the ratio of these cells to the total number of cells that displayed GUS staining, including both nucleus-enriched cells and cells displaying evenly distributed GUS staining between the nucleus and cytoplasm, was calculated. At least 10 seedlings with 20 cells per seedling were analysed for each GUS fusion under both the MS and ACC conditions.

### Nuclear protein extraction

Nuclear proteins were extracted at 4°C using a CelLytic PN Extraction Kit (Sigma-Aldrich) as described previously [67] with minor modifications. After the samples grown on MS medium for 5-days were treated with 50 µM CHX and 5 µM MG132 (to exclude *de novo* protein production and degradation during the process), supplemented with or without 25 µM ACC, in the light for 15 h, the seedlings (3 g) were ground to a fine powder in liquid nitrogen using a precooled mortar and pestle. Next, 15 mL of 1× NIB were mixed with the samples. The suspension was passed through a 100-µm filter mesh into 50-mL tubes. The organelles in the tubes, including the nuclei, were pelleted by centrifugation at 1,200 *g* for 10 min, and the supernatant, including the cytoplasmic fraction, was collected and analysed as the soluble fraction. The pellet was completely resuspended in 1 mL of 1× NIBA (NIB buffer containing a protease inhibitor cocktail), and the organelle membranes were lysed by adding 10% Triton X-100 to a final concentration of 0.3%. To produce a semi-pure nuclear preparation, the lysates were applied to a 0.8-mL cushion of 1.5 M sucrose in 1× NIBA in 1.5-mL tubes. After centrifugation at 12,000 *g* for 10 min, the upper phase and sucrose cushion were removed, and the pellet was washed twice with NIBA buffer. The nuclear pellet was resuspended in 50 µL of nuclear extraction buffer and vortexed for 30 min. The insoluble material was removed by centrifugation at 12,000 *g* for 10 min. The final nuclear protein fraction was transferred to a new tube and stored at −80°C.

## Supporting Information

Figure S1 ACC did not significantly promote the hypocotyl-elongation of *hy5* mutants. (**A**) Morphological observations and (**B**) statistical analyses of hypocotyl length. The images were taken after 5 days of incubation in MS medium supplemented with or without 10 µM ACC. The data indicate the mean values plus the SD from three independent experiments with approximately 30 seedlings. *P*-values (ACC treatment vs. non-treatment) were determined with a two-tailed Student's *t*-test assuming equal variances (*P<0.05).
(TIF)

Figure S2 HY5 negatively regulates ethylene-promoted hypocotyl elongation. (**A**) Morphological observations and (**B**) statistical analyses of hypocotyl length. The images were taken after 5 days of incubation in MS medium supplemented with or without 10 µM ACC. The data indicate the mean values plus the SD from three independent experiments with approximately 30 seedlings. *P*-values (ACC treatment vs. non-treatment) were determined with a two-tailed Student's *t*-test assuming equal variances (*P<0.05).
(TIF)

Figure S3 Ethylene does not transcriptionally regulate *HY5* expression. The expression of *HY5* and its homolog *HYH* in Col-0, the *eto* ethylene overproduction mutants, and the ethylene signalling mutants *ein2* and *ein3-1*. *HY5* transcript levels were quantified by qPCR relative to *TUB4*. The transcript levels of each gene in Col-0 were set to 1. Each value shown is the mean ± SD of three independent biological determinations.
(TIF)

Figure S4 Identification of the transgenic lines of HY5-ΔN77. Identification of the transgenic lines of HY5-ΔN77 driven by the CaMV35S promoter by qPCR with the corresponding primers designed in the N- and C- terminal of *HY5* (*HY5-N*, Ser13-Ala63 and *HY5-C*, Arg84-Gly157). The transcript levels of the gene in Col-0 were set to 1. Each value shown is the mean ± SD of three independent biological determinations.
(TIF)

Figure S5 COP1 functions genetically downstream of ethylene signalling to mediate hypocotyl growth. (**A**) Morphological observations and (**B**) statistical analyses of hypocotyl length. The images were taken after 5 days of incubation in MS medium supplemented with or without 10 µM ACC. The data indicate the mean values plus the SD from three independent experiments with approximately 30 seedlings for each genotype. *P*-values (ACC treatment vs. non-treatment) were determined with a two-tailed Student's *t*-test assuming equal variances (*P<0.05).
(TIF)

Figure S6 The COP1-HY5 complex affects the expression of ethylene-responsive genes. The transcript levels of ethylene-responsive genes in *cop1-4* and *hy5* were quantified by qPCR relative to *TUB4*. The transcript levels of each gene in Col-0 were set to 1. Each value shown is the mean ± SD of three independent biological determinations.
(TIF)

Figure S7 The effect of ethylene on root growth is independent of COP1-HY5. Statistical analyses of root length after 5 days of incubation in MS medium supplemented with or without 10 µM ACC in ethylene signalling mutants (**A**) and in different *COP1* genotypes (**B**). The data indicate the mean values plus the SD from three independent experiments with approximately 30 seedlings. *P*-values (ACC treatment vs. non-treatment) were determined with a two-tailed Student's *t*-test assuming equal variances (*P<0.05).
(TIF)

Figure S8 The ACC-promoted movement of COP1 into the nucleus is not due to circadian rhythms. Statistical summaries of GUS-COP1 localisation in the hypocotyl under different growth conditions. The degree of nuclear enrichment of GUS staining is shown as the percentage of cells with nucleus-enriched GUS relative to the total number of GUS-stained hypocotyl cells. At least 100 cells were counted for each sample. The GUS-COP1 transgenic seedlings were first grown on MS for 4 days and then grown under continuous white light (50 µmol/m^2^s) for another 36 or 48 h with or without 25 µM ACC.
(TIF)

Figure S9 Ethylene enhances COP1 nuclear-enriched localisation. (**A, C, E**) Images and (**B, D, F**) statistical summaries of GUS-COP1 localisation in the hypocotyls (**A–D**) and root (**E, F**) under different growth conditions. The degree of nuclear enrichment of GUS staining is shown as the percentage of cells with nucleus-enriched GUS relative to the total number of GUS-stained hypocotyl cells. At least 100 cells were counted for each sample. GUS-COP1 transgenic seedlings were grown under long-day conditions (16-h light/8-h dark; labelled “16L/8D”) or in the dark (labelled “Dark”) with or without 10 µM ACC for 5 days.
(TIF)

Table S1 The primers used in this study.
(DOC)
