# Supplementary figures and images for: Ethylene Promotes Hypocotyl Growth and HY5 Degradation by Enhancing the Movement of COP1 to the Nucleus in the Light
Source: PLoS Genet. 2013 Dec 12;9(12):e1004025. doi: 10.1371/journal.pgen.1004025 (PMC3861121; doi:10.1371/journal.pgen.1004025)

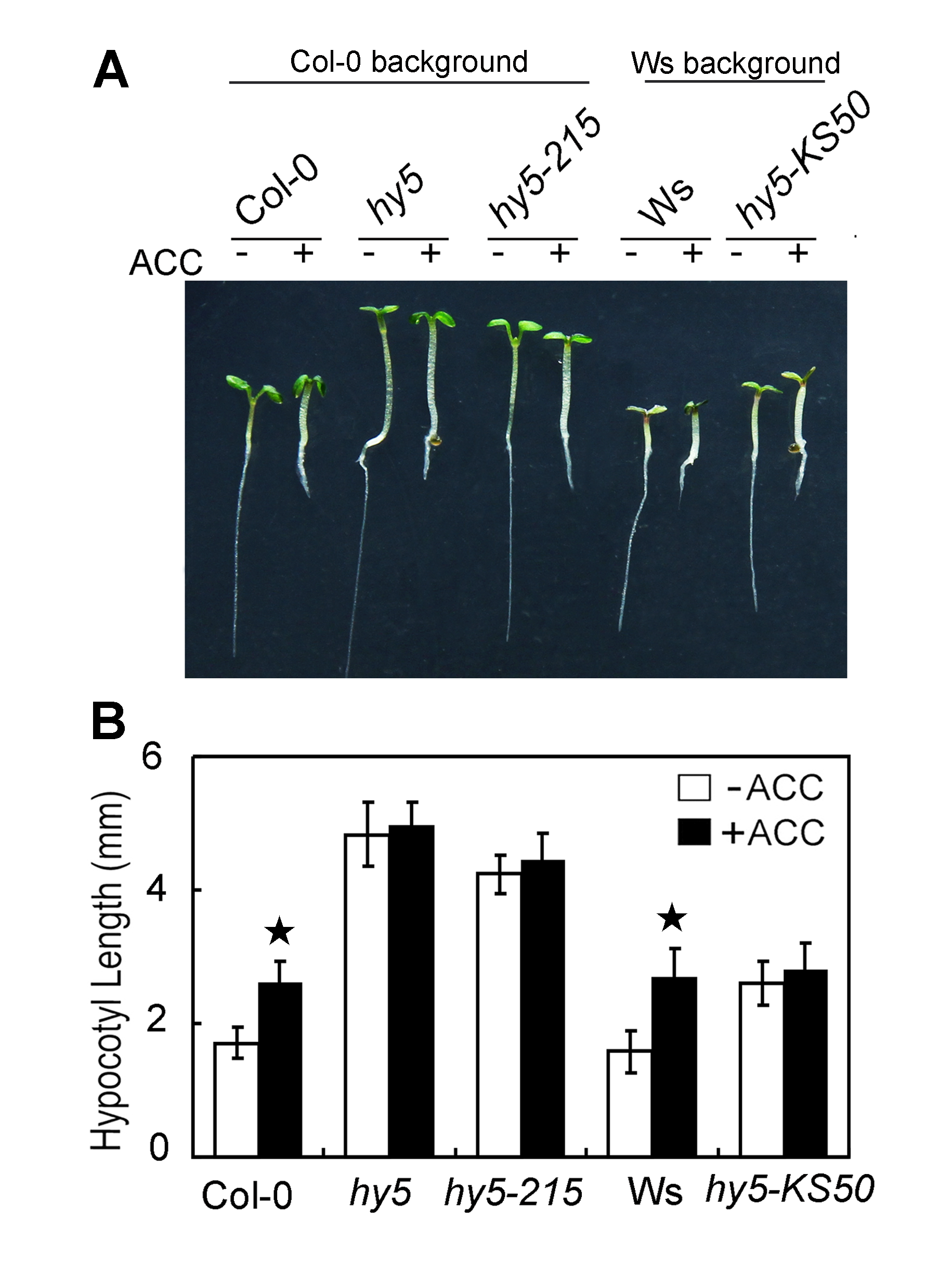

Supplement: Figure S1 — ACC did not significantly promote the hypocotyl-elongation of hy5 mutants. (A) Morphological observations and (B) statistical analyses of hypocotyl length. The images were taken after 5 days of incubation in MS medium supplemented with or without 10 µM ACC. The data indicate the mean values plus the SD from three independent experiments with approximately 30 seedlings. P-values (ACC treatment vs. non-treatment) were determined with a two-tailed Student's t-test assuming equal variances (*P<0.05). (TIF) [file pgen.1004025.s001.tif]

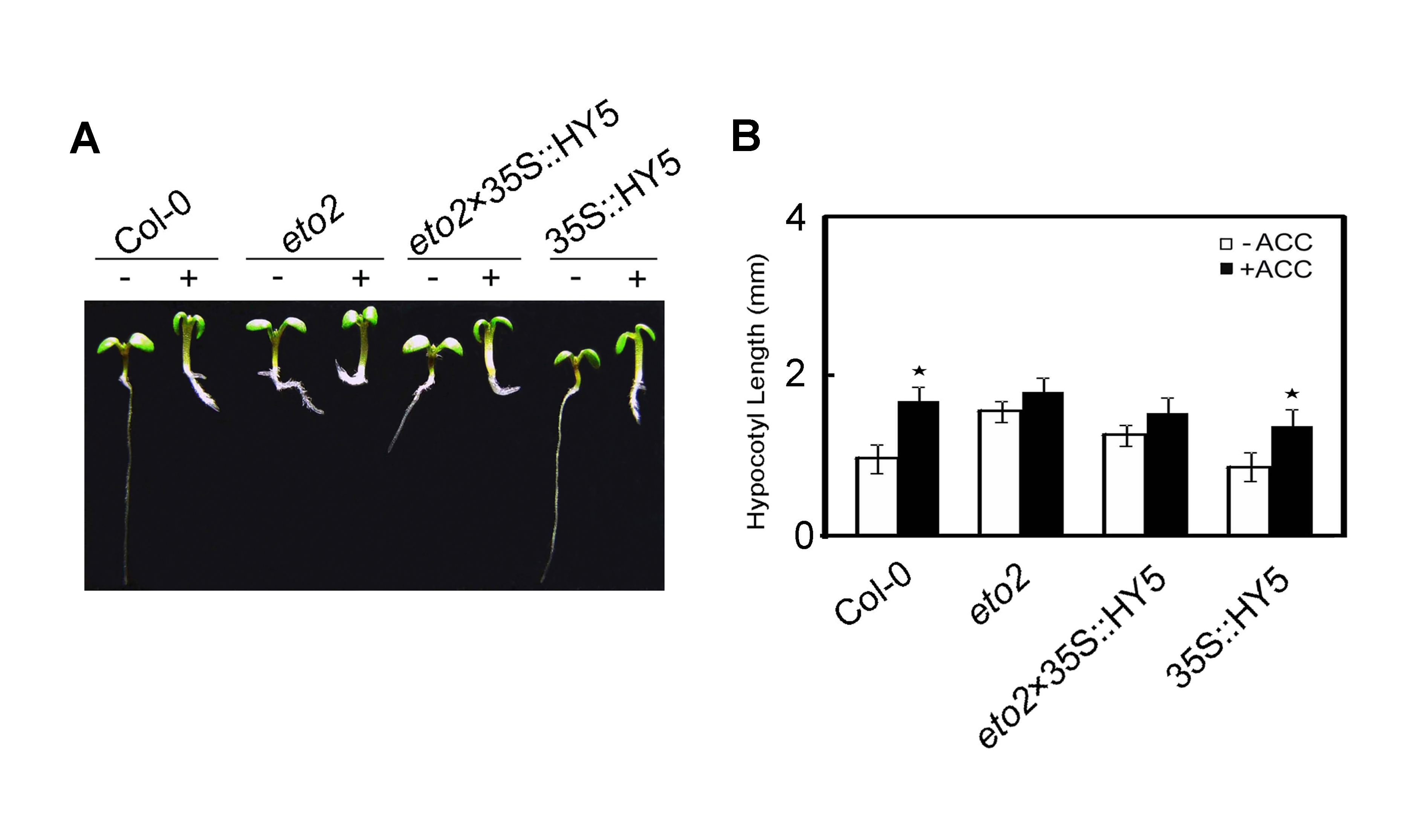

Supplement: Figure S2 — HY5 negatively regulates ethylene-promoted hypocotyl elongation. (A) Morphological observations and (B) statistical analyses of hypocotyl length. The images were taken after 5 days of incubation in MS medium supplemented with or without 10 µM ACC. The data indicate the mean values plus the SD from three independent experiments with approximately 30 seedlings. P-values (ACC treatment vs. non-treatment) were determined with a two-tailed Student's t-test assuming equal variances (*P<0.05). (TIF) [file pgen.1004025.s002.tif]

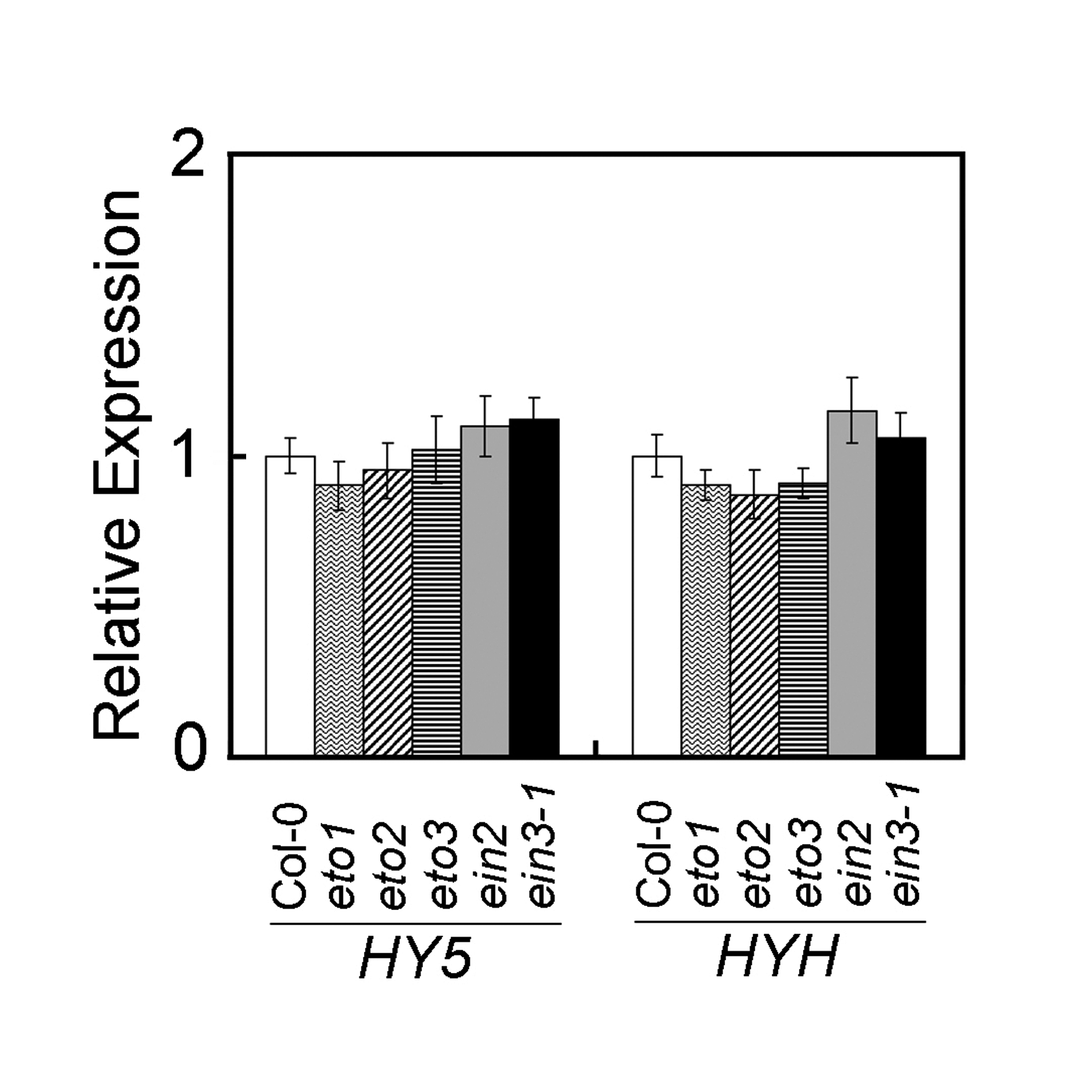

Supplement: Figure S3 — Ethylene does not transcriptionally regulate HY5 expression. The expression of HY5 and its homolog HYH in Col-0, the eto ethylene overproduction mutants, and the ethylene signalling mutants ein2 and ein3-1. HY5 transcript levels were quantified by qPCR relative to TUB4. The transcript levels of each gene in Col-0 were set to 1. Each value shown is the mean ± SD of three independent biological determinations. (TIF) [file pgen.1004025.s003.tif]

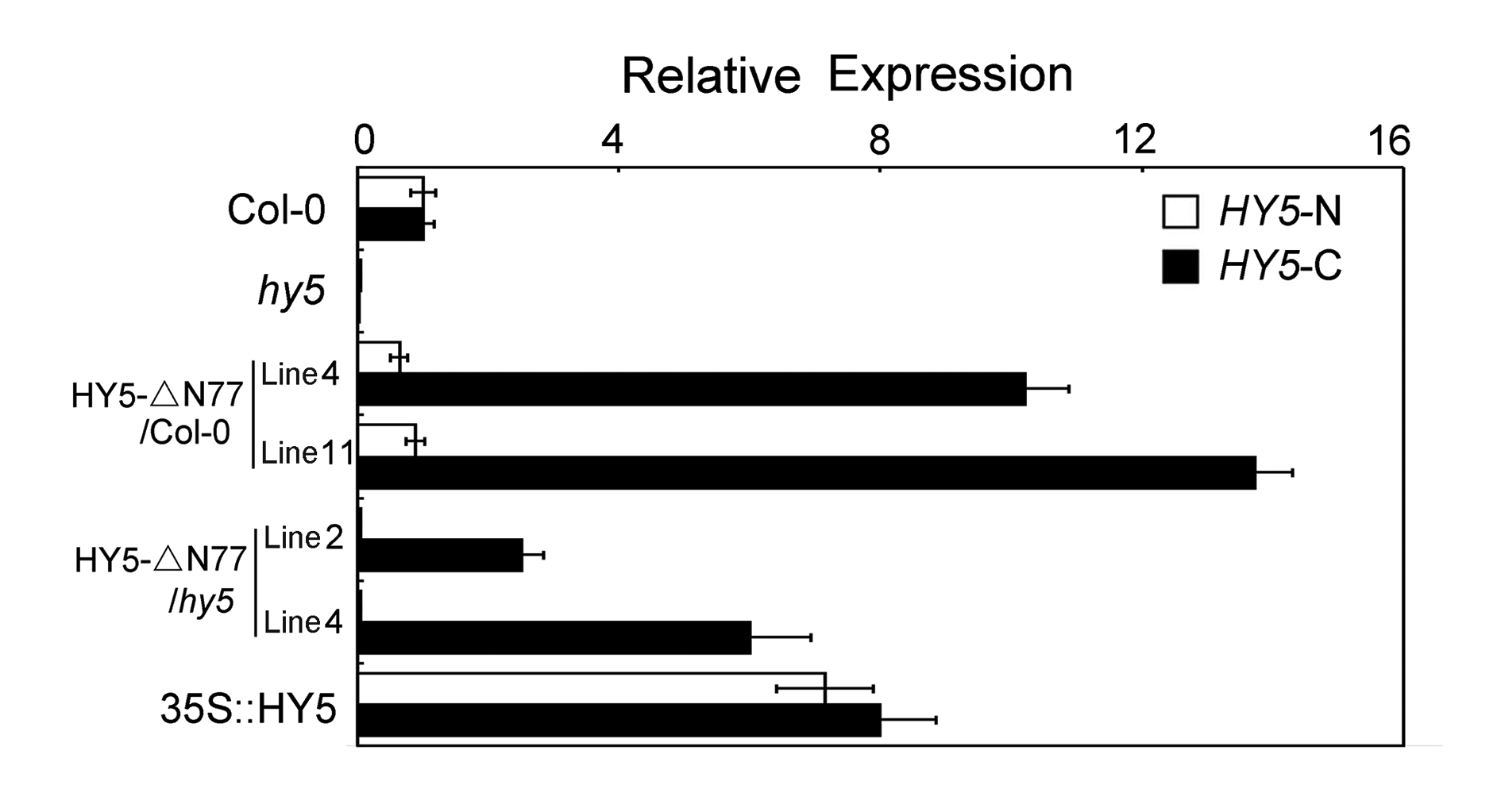

Supplement: Figure S4 — Identification of the transgenic lines of HY5-ΔN77. Identification of the transgenic lines of HY5-ΔN77 driven by the CaMV35S promoter by qPCR with the corresponding primers designed in the N- and C- terminal of HY5 (HY5-N, Ser13-Ala63 and HY5-C, Arg84-Gly157). The transcript levels of the gene in Col-0 were set to 1. Each value shown is the mean ± SD of three independent biological determinations. (TIF) [file pgen.1004025.s004.tif]

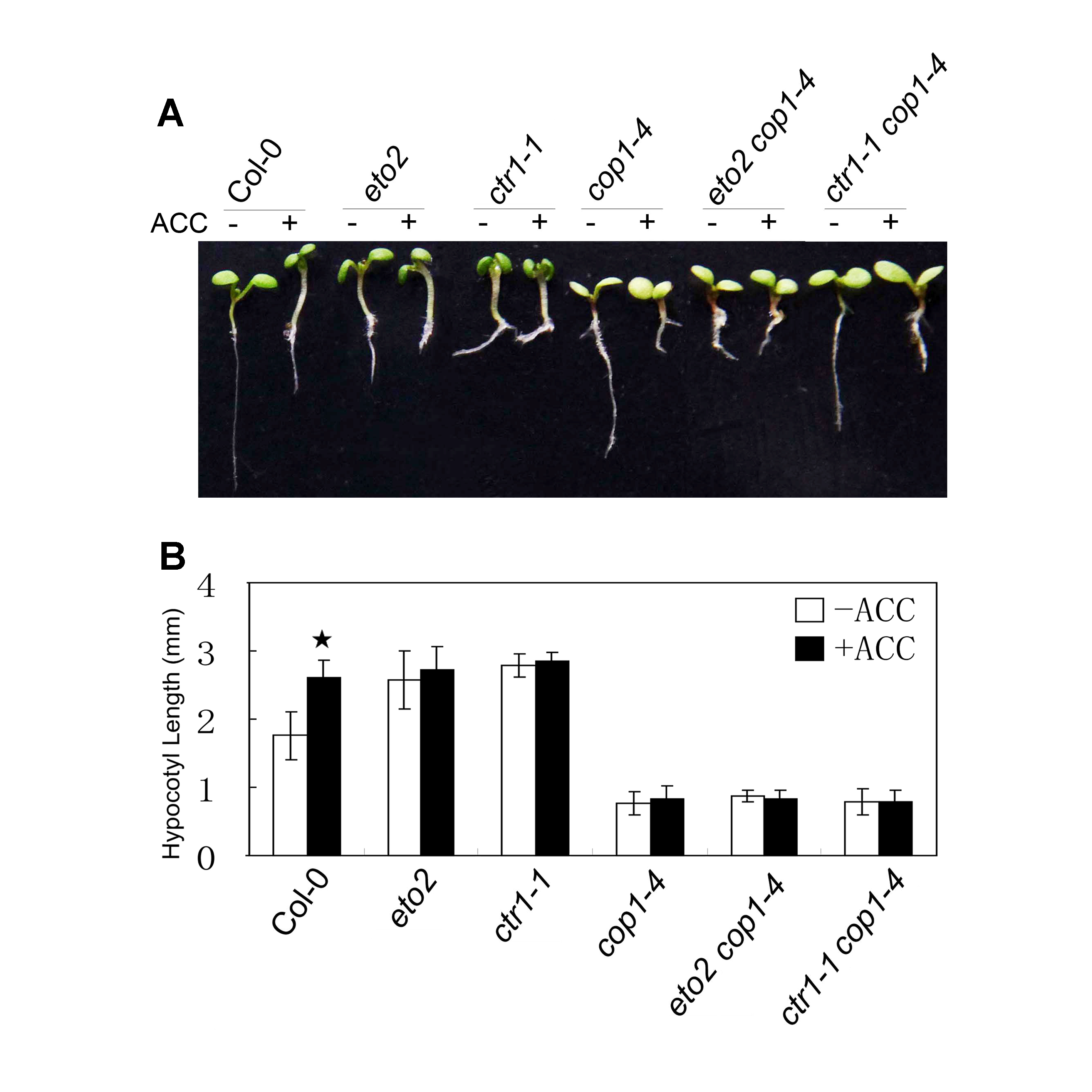

Supplement: Figure S5 — COP1 functions genetically downstream of ethylene signalling to mediate hypocotyl growth. (A) Morphological observations and (B) statistical analyses of hypocotyl length. The images were taken after 5 days of incubation in MS medium supplemented with or without 10 µM ACC. The data indicate the mean values plus the SD from three independent experiments with approximately 30 seedlings for each genotype. P-values (ACC treatment vs. non-treatment) were determined with a two-tailed Student's t-test assuming equal variances (*P<0.05). (TIF) [file pgen.1004025.s005.tif]

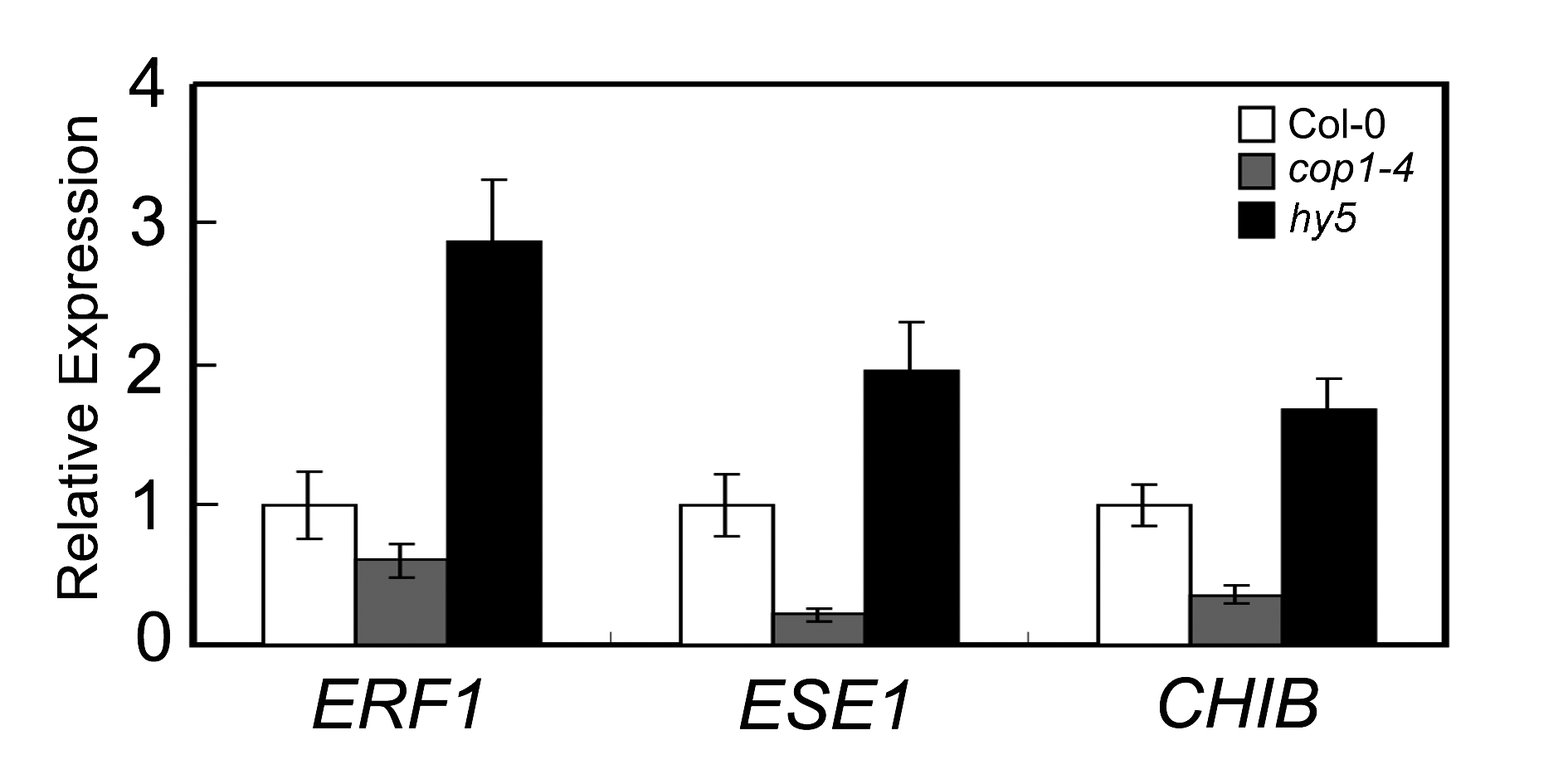

Supplement: Figure S6 — The COP1-HY5 complex affects the expression of ethylene-responsive genes. The transcript levels of ethylene-responsive genes in cop1-4 and hy5 were quantified by qPCR relative to TUB4. The transcript levels of each gene in Col-0 were set to 1. Each value shown is the mean ± SD of three independent biological determinations. (TIF) [file pgen.1004025.s006.tif]

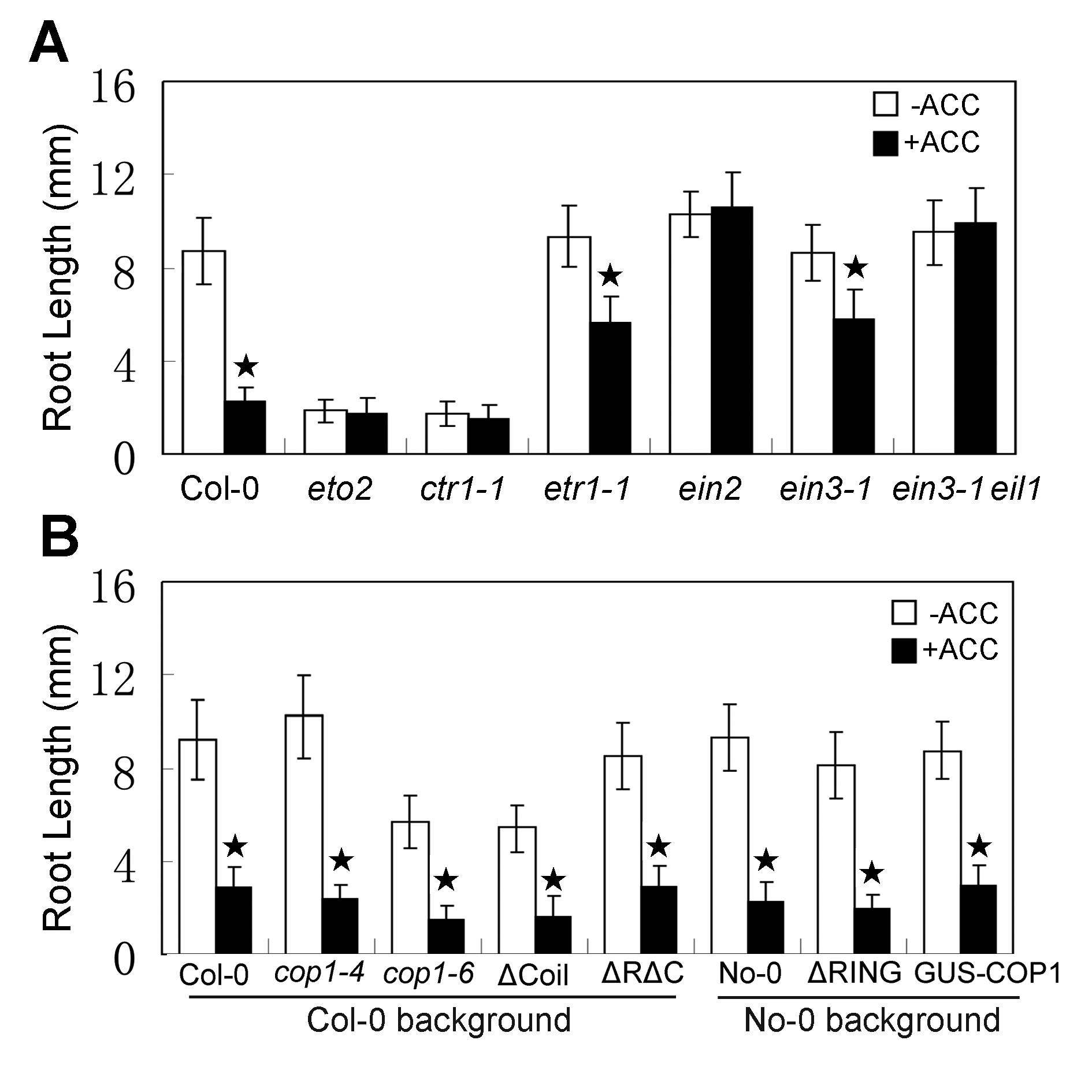

Supplement: Figure S7 — The effect of ethylene on root growth is independent of COP1-HY5. Statistical analyses of root length after 5 days of incubation in MS medium supplemented with or without 10 µM ACC in ethylene signalling mutants (A) and in different COP1 genotypes (B). The data indicate the mean values plus the SD from three independent experiments with approximately 30 seedlings. P-values (ACC treatment vs. non-treatment) were determined with a two-tailed Student's t-test assuming equal variances (*P<0.05). (TIF) [file pgen.1004025.s007.tif]

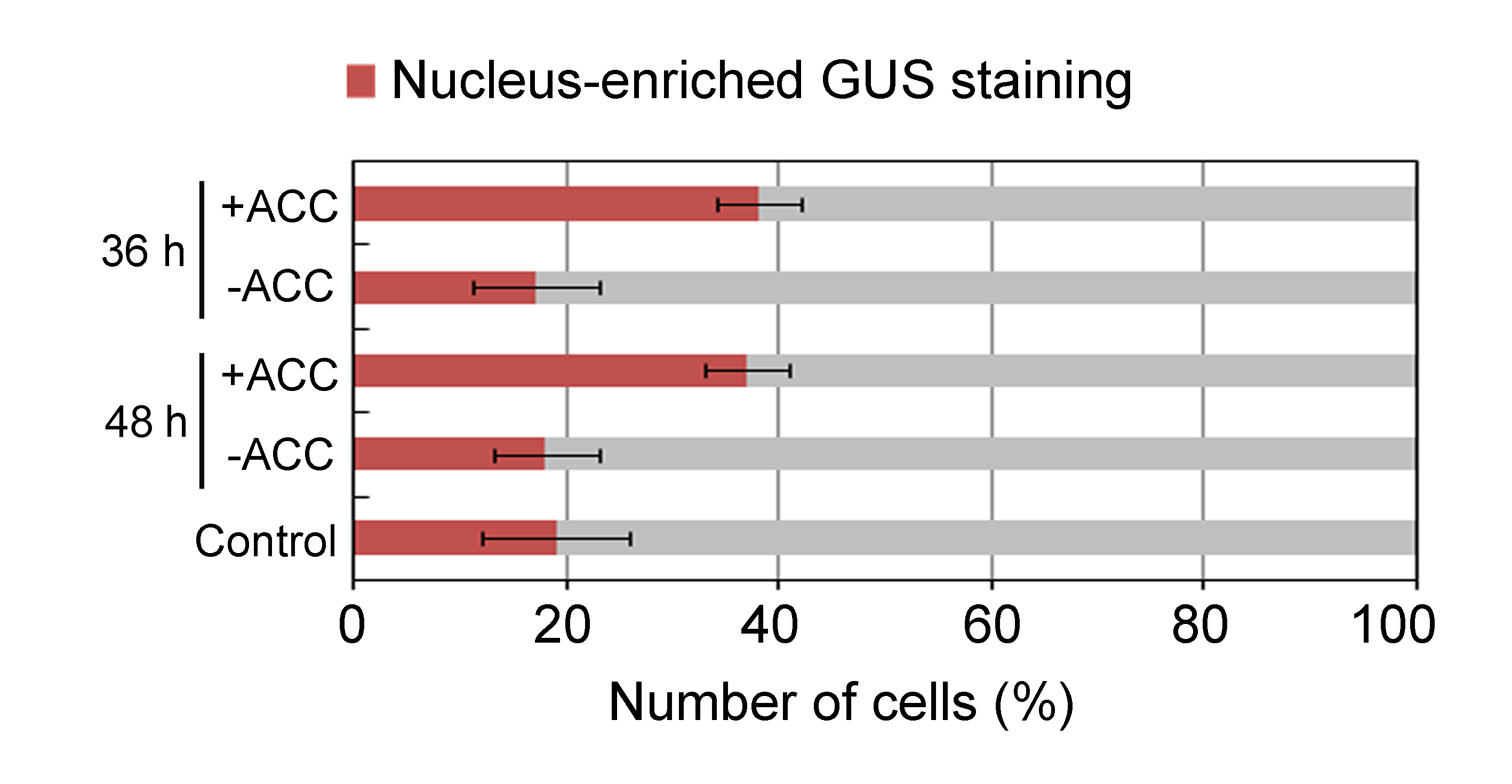

Supplement: Figure S8 — The ACC-promoted movement of COP1 into the nucleus is not due to circadian rhythms. Statistical summaries of GUS-COP1 localisation in the hypocotyl under different growth conditions. The degree of nuclear enrichment of GUS staining is shown as the percentage of cells with nucleus-enriched GUS relative to the total number of GUS-stained hypocotyl cells. At least 100 cells were counted for each sample. The GUS-COP1 transgenic seedlings were first grown on MS for 4 days and then grown under continuous white light (50 µmol/m2s) for another 36 or 48 h with or without 25 µM ACC. (TIF) [file pgen.1004025.s008.tif]

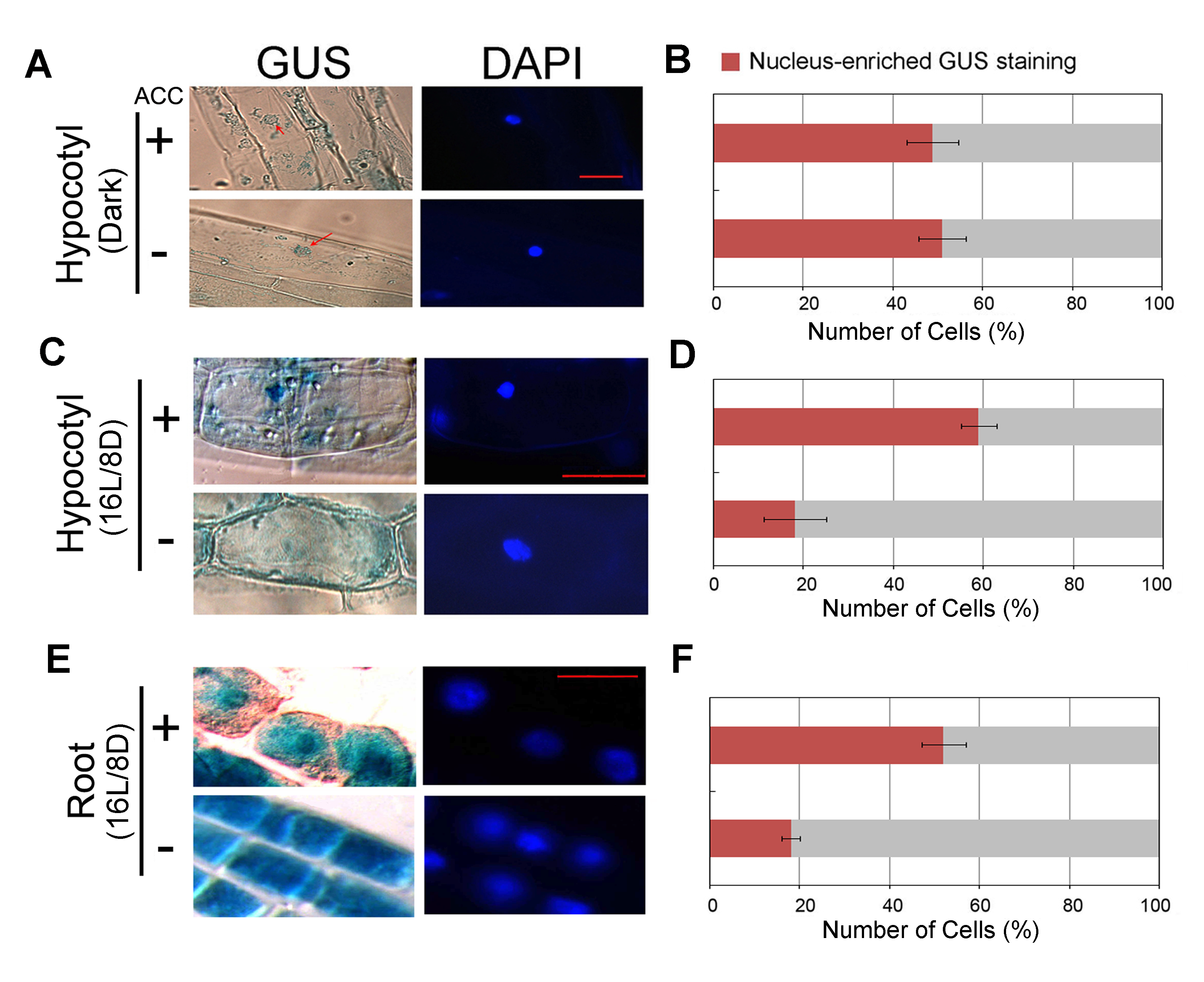

Supplement: Figure S9 — Ethylene enhances COP1 nuclear-enriched localisation. (A, C, E) Images and (B, D, F) statistical summaries of GUS-COP1 localisation in the hypocotyls (A–D) and root (E, F) under different growth conditions. The degree of nuclear enrichment of GUS staining is shown as the percentage of cells with nucleus-enriched GUS relative to the total number of GUS-stained hypocotyl cells. At least 100 cells were counted for each sample. GUS-COP1 transgenic seedlings were grown under long-day conditions (16-h light/8-h dark; labelled “16L/8D”) or in the dark (labelled “Dark”) with or without 10 µM ACC for 5 days. (TIF) [file pgen.1004025.s009.tif]
