# Supplementary material for: Ethylene Promotes Hypocotyl Growth and HY5 Degradation by Enhancing the Movement of COP1 to the Nucleus in the Light
Source: PLoS Genet. 2013 Dec 12;9(12):e1004025. doi: 10.1371/journal.pgen.1004025 (PMC3861121; doi:10.1371/journal.pgen.1004025)
Supplement: Table S1 — The primers used in this study. (DOC) [file pgen.1004025.s010.doc]

**Supplemental Table 1**: The primers used in this study

| ***Primer name*** | **Oligo nucleotide (5’ – 3’ )** |
| --- | --- |
| **qPCR primers** |  |
| *TUB4*-F | CGAAAACGCTGACGAGTGTA |
| *TUB4*-R | CCTTGGGAATGGGATAAGGT |
| *HY5-*F | CTGAAGAACACAACAGGAAACAAG |
| *HY5-*R | TTGCAATATTAGCTCTCACATCCC |
| *HYH-F* | GAGTTACAGAACAACAATGACC |
| *HYH-R*  *ERF1-F*  *ERF1-R*  *ESE1-F*  *ESE1-R*  *CHIB-F*  *CHIB-R*  *HY5N-F*  *HY5N-R*  *HY5C-F*  *HY5C-R* | CATACAACACTGAACAATGG  GAGGAAACACTCGATGAGACG  GGAGCGGTGATCAAAGTCAC  GAGTATCAAATGATGAAGGACGGT  TAGCGGTTTGCGTCGTTACAA  TCCACACTCCAATCCACCGT  CTCCCAAACCGTCTTGCCAT  TCTTTACCATCAAGCAGCG  TCGCCGATCCAGATTCTCT  GGCGGAGAAAGAGAACAAG  CCTCTCTTGTTTCCTGTTGTG |
| **Mutants screened primers** | |
| *eto2-*F | GAAGCAGAGCTTGACCTCTGG |
| *eto2-*R | GTTCGTCGTCTCAAACTTAGGG |
| *ctr1-1-*F | ACTCCTCAGTTTGTCTTGAAGTTTCAGGT |
| *ctr1-1-*R | ACTATTTAGCTTCCATTGGAAATAGGACC |
| **T-DNA screening primers** | |
| *T-DNA_*LBb1.3 | ATTTTGCCGATTTCGGAAC |
| *hy5*-LP | ATTCCTTCCCAAAATGTCTCG |
| *hy5*-RP | ATGCGAGTGAATGACCATTTC |
